# Supplementary material for: Patient groups in Rheumatoid arthritis identified by deep learning respond differently to biologic or targeted synthetic DMARDs
Source: PLoS Comput Biol. 2023 Jun 2;19(6):e1011073. doi: 10.1371/journal.pcbi.1011073 (PMC10266686; doi:10.1371/journal.pcbi.1011073)
Supplement: S1 Text — (DOC) [file pcbi.1011073.s001.doc]

# Supplementary Text S1: More information on Swiss Clinical Quality Management in Rheumatic Diseases (SCQM) registry

Patients come from a wide range of settings (i.e. private practices as well as academic centres) and are usually enrolled prior to the initiation of a b/tsDMARD to allow its nationwide monitoring. RA diagnoses are made by board certified rheumatologists. Follow-up for the SCQM registry involves annual physical examination (i.e., tender joint count, swollen joint count), laboratory tests (i.e., esr), disease activity scores (e.g., DAS28), and several auto-evaluation forms (e.g. health assessment questionnaire [HAQ]). Clinical information of a patient is updated one to four times every year, or every time a patient has a change in antirheumatic therapy. Changes in antirheumatic therapy are captured by the physicians who enters start and stop dates. If a treatment stop occurs the physician can enter the stop reason “non-response”, “adverse event”, “remission”, or “other reason”. Biologic or targeted synthetic disease modifying antirheumatic drug (b/tsDMARD) prescriptions of the same agent were combined as one continuous treatment spell if the gap between stop and start dates were ≤31 days. This allowed for a 1-months grace period between a start and stop of a certain b/tsDMARD which may not reflect a true treatment interruption.
