# Supplementary material for: Patient groups in Rheumatoid arthritis identified by deep learning respond differently to biologic or targeted synthetic DMARDs
Source: PLoS Comput Biol. 2023 Jun 2;19(6):e1011073. doi: 10.1371/journal.pcbi.1011073 (PMC10266686; doi:10.1371/journal.pcbi.1011073)
Supplement: S2 Text — (DOC) [file pcbi.1011073.s002.doc]

# Supplementary Text S2: Clustering - Deep Neural Network Architecture and Training

The AnyNets-Autoencoder uses a set of encoder modules ɸ^i^ for all available (non-missing) input features i of a patient (e.g. sex, age, esr, etc.) to project all non-missing features to a latent space, as described in Kalweit et al. (1). The set of latent representations of the features is pooled by the sum-operation to create a combined vector of fixed length, describing the information available for the patient. The combined vector is fed into an autoencoder module with a latent bottleneck representation **z**, which is a low-dimensional vector representing the patient. The output of the module is fed into a set of decoder modules ꭓ^i^ for the same features as mentioned above in order to learn reconstructions of the respective input features. In this work, we use encoder and decoder networks with two layers and a hidden dimension 64, a hidden dimension of 1024 for the autoencoder module, and a bottleneck of size 10. The network is pre-trained to reconstruct the inputs for 25’000 iterations and trained on the deep embedded clustering (DEC) loss until the label assignment change (in percentage) between two consecutive updates for target distribution is smaller than 10^-4^, as described in Guo et al. (2). As optimizer, we used Adam as described in Kingma et al. (3) with a learning rate of 10^-3^.

**References:**

1. Kalweit M, Kalweit G, Boedecker J. AnyNets: Adaptive Deep Neural Networks for Medical Data with Missing Values. Accepted at IJCAI 2021 Workshop on Artificial Intelligence for Function, Disability, and Health. 2nd Workshop on Artificial Intelligence for Function, Disability, and Health. 2021;

2. Guo X, Gao L, Liu X, Yin J. Improved deep embedded clustering with local structure preservation. IJCAI International Joint Conference on Artificial Intelligence. 2017;0:1753–9.

3. Kingma DP, Ba J. Adam: A Method for Stochastic Optimization. 2014 Dec 22; Available from: http://arxiv.org/abs/1412.6980
