# Supplementary material for: Patient groups in Rheumatoid arthritis identified by deep learning respond differently to biologic or targeted synthetic DMARDs
Source: PLoS Comput Biol. 2023 Jun 2;19(6):e1011073. doi: 10.1371/journal.pcbi.1011073 (PMC10266686; doi:10.1371/journal.pcbi.1011073)
Supplement: S3 Text — (DOC) [file pcbi.1011073.s003.doc]

# S3 Text: Example of approach how cluster groups were formed

All columns were put side by side (in this example only a fraction of columns and pre-selected characteristics are seen). Most extreme values were marked. In a further step, columns were re-shuffled to put similar columns next to each other. Groups were formed.

Selected patient characteristics of selected clusters with extreme values marked in red

Selected patient characteristics of selected clusters with extreme values marked (in red) and columns re-shuffled to put similar clusters next to each other

Patients with highest use of csDMARDs and prednisone, a tendency towards no family history of RD and a higher proportion of women

Men

Seronegative patients with lowest use of prednisone and a tendency towards a higher proportion of women

Seronpositive women with a rather high disease burden and long disease duration

Patients with a rather low disease burden and a tendency towards seropositivy and a higher proportion of women
