# Supplementary material for: Patient groups in Rheumatoid arthritis identified by deep learning respond differently to biologic or targeted synthetic DMARDs
Source: PLoS Comput Biol. 2023 Jun 2;19(6):e1011073. doi: 10.1371/journal.pcbi.1011073 (PMC10266686; doi:10.1371/journal.pcbi.1011073)
Supplement: S4 Text — (DOC) [file pcbi.1011073.s004.doc]

# S4 Text: Comparative effectiveness analyses in each of the 24 clusters

The following tables present comparative effectiveness analyses of each 24 individual clusters (i.e. 3, 4, and 5 clusters run by two different specifications) allocated by suggested groups according to patient characteristics.

The following supplementary tables show results of comparative effectiveness analyses in clusters grouped together to make up group 1 (i.e. clusters with high use of conventional synthetic DMARDs and prednisone). The majority of clusters suggest a good response to tocilizumab.

**Supplementary Table.** Comparative effectiveness analyses in the first cluster of group 1 (S2 Table)

|  | **Treatment disc. due to non-response** | **Crude HR**  **(95% CI)** | **20% DAS28-esr reduction** | **Crude HR**  **(95% CI)** |
| --- | --- | --- | --- | --- |
| **TNF-inhibitor** | 133 | **Ref 1.00** | 69 | **Ref 1.00** |
| Adalimumab | 52 | Ref 1.00 | 25 | Ref 1.00 |
| Certolizumab | 9 | 2.55 (1.25-5.17) | 5 | 2.64 (1.01-6.90) |
| Etanercept | 38 | 0.82 (0.54-1.25) | 20 | 0.91 (0.50-1.63) |
| Golimumab | 20 | 1.79 (1.02-2.85) | 11 | 1.93 (0.95-3.92) |
| Infliximab | 14 | 0.69 (0.38-1.24) | 8 | 0.84 (0.38-1.86) |
| **Non-TNF-inh.** | 18 | 0.66 (0.40-1.08) | 14 | 1.07 (0.60-1.91) |
| Abatacept | 9 | 0.75 (0.37-1.52) | 3 | NA |
| Rituximab | 2 | NA | 2 | NA |
| Tocilizumab | 7 | 0.76 (0.34-1.67) | 9 | 2.65 (1.24-5.69) |
| **JAK-inhibitor** | 2 | NA | 4 | **NA** |
| Baricitinib | 0 | NA | 0 | NA |
| Tofacitinib | 2 | NA | 4 | NA |

CI: confidence interval; Disc.: discontinuation; esr: erythrocyte sedimentation rate; HR: hazard radio; inh.: inhibitor; JAK: janus kinase; TNF: tumor necrosis factor alpha;

Results in red color are significant results.

**Supplementary Table.** Comparative effectiveness analyses in the second cluster of group 1 (S2 Table)

|  | **Treatment disc. due to non-response** | **Crude HR**  **(95% CI)** | **20% DAS28-esr reduction** | **Crude HR**  **(95% CI)** |
| --- | --- | --- | --- | --- |
| **TNF-inhibitor** | 96 | Ref 1.00 | 47 | Ref 1.00 |
| Adalimumab | 40 | Ref 1.00 | 17 | Ref 1.00 |
| Certolizumab | 8 | 2.85 (1.33-6.09) | 4 | NA |
| Etanercept | 27 | 0.82 (0.50-1.34) | 15 | 1.11 (0.56-2.23) |
| Golimumab | 10 | 1.28 (0.64-2.56) | 5 | 1.55 (0.57-4.20) |
| Infliximab | 11 | 0.69 (0.36-1.35) | 6 | 0.89 (0.35-2.25) |
| **Non-TNF-inh.** | 14 | 0.61 (0.35-1.07) | 11 | 1.06 (0.55-2.05) |
| Abatacept | 8 | 0.74 (0.35-1.59) | 3 | NA |
| Rituximab | 1 | NA | 2 | NA |
| Tocilizumab | 5 | 0.76 (0.30-1.92) | 6 | 2.67 (1.05-6.78) |
| **JAK-inhibitor** | 3 | NA | 3 | NA |
| Baricitinib | 0 | NA | 0 | NA |
| Tofacitinib | 3 | NA | 3 | NA |

CI: confidence interval; Disc.: discontinuation; esr: erythrocyte sedimentation rate; HR: hazard radio; inh.: inhibitor; JAK: janus kinase; TNF: tumor necrosis factor alpha;

Results in red color are significant results.

**Supplementary Table.** Comparative effectiveness analyses in the third cluster of group 1 (S2 Table)

|  | **Treatment disc. due to non-response** | **Crude HR**  **(95% CI)** | **20% DAS28-esr reduction** | **Crude HR**  **(95% CI)** |
| --- | --- | --- | --- | --- |
| **TNF-inhibitor** | 80 | Ref 1.00 | 39 | Ref 1.00 |
| Adalimumab | 32 | Ref 1.00 | 12 | Ref 1.00 |
| Certolizumab | 7 | 2.91 (1.29-6.60) | 4 | NA |
| Etanercept | 24 | 0.91 (0.54-1.55) | 13 | 1.34 (0.61-2.93) |
| Golimumab | 9 | 1.67 (0.80-3.50) | 4 | NA |
| Infliximab | 8 | 0.64 (0.30-1.40) | 6 | 1.34 (0.50-3.56) |
| **Non-TNF-inh.** | 10 | 0.60 (0.31-1.15) | 8 | 1.08 (0.51-2.30) |
| Abatacept | 6 | 0.79 (0.33-1.88) | 3 | NA |
| Rituximab | 1 | NA | 1 | NA |
| Tocilizumab | 3 | NA | 4 | NA |
| **JAK-inhibitor** | 2 | NA | 3 | NA |
| Baricitinib | 0 | NA | 0 | NA |
| Tofacitinib | 2 | NA | 3 | NA |

CI: confidence interval; Disc.: discontinuation; esr: erythrocyte sedimentation rate; HR: hazard radio; inh.: inhibitor; JAK: janus kinase; TNF: tumor necrosis factor alpha;

Results in red color are significant results.

**Supplementary Table.** Comparative effectiveness analyses in the fourth cluster of group 1 (S2 Table)

|  | **Treatment disc. due to non-response** | **Crude HR**  **(95% CI)** | **20% DAS28-esr reduction** | **Crude HR**  **(95% CI)** |
| --- | --- | --- | --- | --- |
| **TNF-inhibitor** | 127 | Ref 1.00 | 66 | Ref 1.00 |
| Adalimumab | 45 | Ref 1.00 | 21 | Ref 1.00 |
| Certolizumab | 5 | 0.97 (0.39-2.46) | 2 | NA |
| Etanercept | 39 | 0.92 (0.60-1.42) | 21 | 1.13 (0.62-2.06) |
| Golimumab | 18 | 1.28 (0.74-2.21) | 10 | 1.54 (0.73-3.27) |
| Infliximab | 20 | 0.79 (0.47-1.34) | 12 | 1.17 (0.58-2.39) |
| **Non-TNF-inh.** | 25 | 0.85 (0.55-1.30) | 15 | 1.04 (0.59-1.82) |
| Abatacept | 12 | 0.86 (0.46-1.63) | 2 | NA |
| Rituximab | 3 | NA | 2 | NA |
| Tocilizumab | 10 | 0.94 (0.47-1.87) | 11 | 2.55 (1.23-5.30) |
| **JAK-inhibitor** | 5 | 0.74 (0.30-1.80) | 5 | 1.45 (0.58-3.60) |
| Baricitinib | 0 | NA | 2 | NA |
| Tofacitinib | 5 | 0.77 (0.31-1.94) | 3 | NA |

CI: confidence interval; Disc.: discontinuation; esr: erythrocyte sedimentation rate; HR: hazard radio; inh.: inhibitor; JAK: janus kinase; TNF: tumor necrosis factor alpha;

Results in red color are significant results.

**Supplementary Table.** Comparative effectiveness analyses in the fifth cluster of group 1 (S2 Table)

|  | **Treatment disc. due to non-response** | **Crude HR**  **(95% CI)** | **20% DAS28-esr reduction** | **Crude HR**  **(95% CI)** |
| --- | --- | --- | --- | --- |
| **TNF-inhibitor** | 39 | Ref 1.00 | 20 | Ref 1.00 |
| Adalimumab | 24 | Ref 1.00 | 6 | Ref 1.00 |
| Certolizumab | 1 | NA | 0 | NA |
| Etanercept | 8 | 0.87 (0.33-2.29) | 9 | 1.88 (0.67-5.28) |
| Golimumab | 5 | 0.11 (0.02-0.80) | 3 | NA |
| Infliximab | 1 | NA | 2 | NA |
| **Non-TNF-inh.** | 8 | 0.71 (0.33-1.51) | 7 | 1.47 (0.62-3.47) |
| Abatacept | 5 | 0.72 (0.27-1.88) | 3 | NA |
| Rituximab | 1 | NA | 1 | NA |
| Tocilizumab | 2 | NA | 3 | NA |
| **JAK-inhibitor** | 1 | NA | 1 | NA |
| Baricitinib | 0 | NA | 0 | NA |
| Tofacitinib | 1 | NA | 1 | NA |

CI: confidence interval; Disc.: discontinuation; esr: erythrocyte sedimentation rate; HR: hazard radio; inh.: inhibitor; JAK: janus kinase; TNF: tumor necrosis factor alpha;

Results in red color are significant results.

The following supplementary tables show results of comparative effectiveness analyses in clusters grouped together to make up group 2 (i.e. clusters of men). The majority of clusters suggest a good response to tocilizumab.

**Supplementary Table.** Comparative effectiveness analyses in the first cluster of group 2 (S3 Table)

|  | **Treatment disc. due to non-response** | **Crude HR**  **(95% CI)** | **20% DAS28-esr reduction** | **Crude HR**  **(95% CI)** |
| --- | --- | --- | --- | --- |
| **TNF-inhibitor** | 71 | Ref 1.00 | 49 | Ref 1.00 |
| Adalimumab | 30 | Ref 1.00 | 16 | Ref 1.00 |
| Certolizumab | 0 | NA | 0 | NA |
| Etanercept | 19 | 0.77 (0.43-1.36) | 15 | 1.21 (0.60-2.44) |
| Golimumab | 11 | 2.33 (1.17-4.65) | 8 | 2.97 (1.27-6.90) |
| Infliximab | 11 | 0.63 (0.32-1.26) | 10 | 1.17 (0.53-2.57) |
| **Non-TNF-inh.** | 11 | 1.39 (0.74-2.62) | 9 | 1.76 (0.86-3.60) |
| Abatacept | 6 | 1.22 (0.51-2.93) | 0 | NA |
| Rituximab | 1 | NA | 3 | NA |
| Tocilizumab | 4 | NA | 6 | 6.78 (2.65-17.35) |
| **JAK-inhibitor** | 2 | NA | 4 | NA |
| Baricitinib | 0 | NA | 0 | NA |
| Tofacitinib | 2 | NA | 4 | NA |

CI: confidence interval; Disc.: discontinuation; esr: erythrocyte sedimentation rate; HR: hazard radio; inh.: inhibitor; JAK: janus kinase; TNF: tumor necrosis factor alpha;

Results in red color are significant results.

**Supplementary Table.** Comparative effectiveness analyses in the second cluster of group 2 (S3 Table)

|  | **Treatment disc. due to non-response** | **Crude HR**  **(95% CI)** | **20% DAS28-esr reduction** | **Crude HR**  **(95% CI)** |
| --- | --- | --- | --- | --- |
| **TNF-inhibitor** | 87 | Ref 1.00 | 53 | Ref 1.00 |
| Adalimumab | 37 | Ref 1.00 | 16 | Ref 1.00 |
| Certolizumab | 1 | NA | 1 | NA |
| Etanercept | 25 | 0.81 (0.49-1.34) | 18 | 1.44 (0.74-2.83) |
| Golimumab | 13 | 2.23 (1.18-4.19) | 8 | 2.95 (1.26-6.88) |
| Infliximab | 11 | 0.54 (0.28-1.06) | 10 | 1.22 (0.56-2.70) |
| **Non-TNF-inh.** | 13 | 1.32 (0.74-2.36) | 11 | 2.03 (1.06-3.90) |
| Abatacept | 7 | 1.25 (0.56-2.81) | 0 | NA |
| Rituximab | 2 | NA | 4 | NA |
| Tocilizumab | 4 | NA | 7 | 8.54 (3.50-20.81) |
| **JAK-inhibitor** | 3 | NA | 6 | 3.76 (1.62-8.80) |
| Baricitinib | 0 | NA | 0 | NA |
| Tofacitinib | 3 | NA | 6 | 5.00 (1.96-12.78) |

CI: confidence interval; Disc.: discontinuation; esr: erythrocyte sedimentation rate; HR: hazard radio; inh.: inhibitor; JAK: janus kinase; TNF: tumor necrosis factor alpha;

Results in red color are significant results.

**Supplementary Table.** Comparative effectiveness analyses in the third cluster of group 2 (S3 Table)

|  | **Treatment disc. due to non-response** | **Crude HR**  **(95% CI)** | **20% DAS28-esr reduction** | **Crude HR**  **(95% CI)** |
| --- | --- | --- | --- | --- |
| **TNF-inhibitor** | 73 | Ref 1.00 | 43 | Ref 1.00 |
| Adalimumab | 30 | Ref 1.00 | 14 | Ref 1.00 |
| Certolizumab | 1 | NA | 1 | NA |
| Etanercept | 22 | 0.89 (0.52-1.55) | 13 | 1.17 (0.55-2.50) |
| Golimumab | 10 | 1.96 (0.96-4.02) | 6 | 2.32 (0.89-6.04) |
| Infliximab | 10 | 0.58 (0.28-1.19) | 9 | 1.19 (0.51-2.75) |
| **Non-TNF-inh.** | 8 | 1.02 (0.49-2.13) | 8 | 1.91 (0.90-4.07) |
| Abatacept | 4 | NA | 0 | NA |
| Rituximab | 2 | NA | 3 | NA |
| Tocilizumab | 2 | NA | 5 | 7.95 (2.85-22.16) |
| **JAK-inhibitor** | 3 | NA | 5 | 4.56 (1.81-11.53) |
| Baricitinib | 0 | NA | 0 | NA |
| Tofacitinib | 3 | NA | 5 | 5.45 (1.96-15.15) |

CI: confidence interval; Disc.: discontinuation; esr: erythrocyte sedimentation rate; HR: hazard radio; inh.: inhibitor; JAK: janus kinase; TNF: tumor necrosis factor alpha;

Results in red color are significant results.

The following supplementary tables show results of comparative effectiveness analyses in clusters grouped together to make up group 3 (i.e. clusters of seronegative patients with a tendency towards low use of prednisone and a higher proportion of women). The majority of clusters suggest a high risk of non-response with golimumab.

**Supplementary Table.** Comparative effectiveness analyses in the first cluster of group 3 (S4 Table)

|  | **Treatment disc. due to non-response** | **Crude HR**  **(95% CI)** | **20% DAS28-esr reduction** | **Crude HR**  **(95% CI)** |
| --- | --- | --- | --- | --- |
| **TNF-inhibitor** | 104 | Ref 1.00 | 63 | Ref 1.00 |
| Adalimumab | 35 | Ref 1.00 | 17 | Ref 1.00 |
| Certolizumab | 2 | NA | 1 | NA |
| Etanercept | 37 | 0.93 (0.59-1.48) | 24 | 1.25 (0.67-2.30) |
| Golimumab | 19 | 2.10 (1.20-3.67) | 12 | 2.88 (1.38-6.04) |
| Infliximab | 11 | 0.63 (0.32-1.25) | 9 | 1.08 (0.48-2.40) |
| **Non-TNF-inh.** | 12 | 1.00 (0.55-1.81) | 9 | 1.33 (0.66-2.67) |
| Abatacept | 7 | 1.93 (0.86-4.33) | 4 | NA |
| Rituximab | 1 | NA | 2 | NA |
| Tocilizumab | 4 | NA | 3 | NA |
| **JAK-inhibitor** | 1 | NA | 6 | 2.40 (1.04-5.60) |
| Baricitinib | 0 | NA | 0 | NA |
| Tofacitinib | 1 | NA | 6 | 3.11 (1.22-7.90) |

CI: confidence interval; Disc.: discontinuation; esr: erythrocyte sedimentation rate; HR: hazard radio; inh.: inhibitor; JAK: janus kinase; TNF: tumor necrosis factor alpha;

Results in red color are significant results.

**Supplementary Table.** Comparative effectiveness analyses in the second cluster of group 3 (S4 Table)

|  | **Treatment disc. due to non-response** | **Crude HR**  **(95% CI)** | **20% DAS28-esr reduction** | **Crude HR**  **(95% CI)** |
| --- | --- | --- | --- | --- |
| **TNF-inhibitor** | 89 | Ref 1.00 | 46 | Ref 1.00 |
| Adalimumab | 28 | Ref 1.00 | 14 | Ref 1.00 |
| Certolizumab | 2 | NA | 1 | NA |
| Etanercept | 34 | 0.97 (0.59-1.59) | 17 | 0.99 (0.49-2.00) |
| Golimumab | 16 | 2.12 (1.15-3.91) | 8 | 2.33 (0.98-5.56) |
| Infliximab | 9 | 0.66 (0.31-1.41) | 6 | 0.89 (0.34-2.30) |
| **Non-TNF-inh.** | 9 | 0.73 (0.37-1.46) | 7 | 1.15 (0.52-2.60) |
| Abatacept | 7 | 2.03 (0.89-4.65) | 4 | NA |
| Rituximab | 0 | NA | 2 | NA |
| Tocilizumab | 2 | NA | 1 | NA |
| **JAK-inhibitor** | 1 | NA | 4 | NA |
| Baricitinib | 0 | NA | 0 | NA |
| Tofacitinib | 1 | NA | 4 | NA |

CI: confidence interval; Disc.: discontinuation; esr: erythrocyte sedimentation rate; HR: hazard radio; inh.: inhibitor; JAK: janus kinase; TNF: tumor necrosis factor alpha;

Results in red color are significant results.

**Supplementary Table.** Comparative effectiveness analyses in the third cluster of group 3 (S4 Table)

|  | **Treatment disc. due to non-response** | **Crude HR**  **(95% CI)** | **20% DAS28-esr reduction** | **Crude HR**  **(95% CI)** |
| --- | --- | --- | --- | --- |
| **TNF-inhibitor** | 129 | Ref 1.00 | 68 | Ref 1.00 |
| Adalimumab | 46 | Ref 1.00 | 19 | Ref 1.00 |
| Certolizumab | 5 | 1.38 (0.55-3.46) | 1 | NA |
| Etanercept | 41 | 0.85 (0.56-1.30) | 25 | 1.27 (0.70-2.31) |
| Golimumab | 24 | 2.15 (1.31-3.52) | 13 | 2.95 (1.46-5.98) |
| Infliximab | 13 | 0.61 (0.33-1.13) | 10 | 1.14 (0.53-2.44) |
| **Non-TNF-inh.** | 16 | 0.98 (0.58-1.65) | 11 | 1.37 (0.72-2.58) |
| Abatacept | 11 | 1.75 (0.91-3.39) | 5 | 1.82 (0.68-4.88) |
| Rituximab | 1 | NA | 2 | NA |
| Tocilizumab | 4 | NA | 4 | NA |
| **JAK-inhibitor** | 2 | NA | 8 | 3.23 (1.55-6.73) |
| Baricitinib | 0 | NA | 0 | NA |
| Tofacitinib | 2 | NA | 8 | 4.21 (1.84-9.63) |

CI: confidence interval; Disc.: discontinuation; esr: erythrocyte sedimentation rate; HR: hazard radio; inh.: inhibitor; JAK: janus kinase; TNF: tumor necrosis factor alpha;

Results in red color are significant results.

**Supplementary Table.** Comparative effectiveness analyses in the fourth cluster of group 3 (S4 Table)

|  | **Treatment disc. due to non-response** | **Crude HR**  **(95% CI)** | **20% DAS28-esr reduction** | **Crude HR**  **(95% CI)** |
| --- | --- | --- | --- | --- |
| **TNF-inhibitor** | 102 | Ref 1.00 | 51 | Ref 1.00 |
| Adalimumab | 33 | Ref 1.00 | 15 | Ref 1.00 |
| Certolizumab | 5 | 2.09 (0.82-5.35) | 1 | NA |
| Etanercept | 33 | 0.89 (0.55-1.45) | 18 | 1.09 (0.55-2.17) |
| Golimumab | 19 | 2.20 (1.25-3.87) | 9 | 2.44 (1.07-5.58) |
| Infliximab | 12 | 0.85 (0.44-1.64) | 8 | 1.25 (0.53-2.94) |
| **Non-TNF-inh.** | 12 | 0.81 (0.45-1.47) | 9 | 1.28 (0.63-2.59) |
| Abatacept | 10 | 1.84 (0.90-3.73) | 5 | 1.98 (0.72-5.45) |
| Rituximab | 0 | NA | 2 | NA |
| Tocilizumab | 2 | NA | 2 | NA |
| **JAK-inhibitor** | 1 | NA | 4 | NA |
| Baricitinib | 0 | NA | 0 | NA |
| Tofacitinib | 1 | NA | 4 | NA |

CI: confidence interval; Disc.: discontinuation; esr: erythrocyte sedimentation rate; HR: hazard radio; inh.: inhibitor; JAK: janus kinase; TNF: tumor necrosis factor alpha;

Results in red color are significant results.

**Supplementary Table.** Comparative effectiveness analyses in the fifth cluster of group 3 (S4 Table)

|  | **Treatment disc. due to non-response** | **Crude HR**  **(95% CI)** | **20% DAS28-esr reduction** | **Crude HR**  **(95% CI)** |
| --- | --- | --- | --- | --- |
| **TNF-inhibitor** | 93 | Ref 1.00 | 45 | Ref 1.00 |
| Adalimumab | 27 | Ref 1.00 | 13 | Ref 1.00 |
| Certolizumab | 4 | NA | 1 | NA |
| Etanercept | 31 | 0.95 (0.57-1.59) | 16 | 1.07 (0.51-2.22) |
| Golimumab | 19 | 2.53 (1.40-4.54) | 8 | 2.38 (0.99-5.75) |
| Infliximab | 12 | 0.93 (0.47-1.83) | 7 | 1.13 (0.45-2.84) |
| **Non-TNF-inh.** | 10 | 0.83 (0.43-1.59) | 6 | 1.06 (0.45-2.47) |
| Abatacept | 8 | 1.58 (0.72-3.47) | 2 | NA |
| Rituximab | 0 | NA | 2 | NA |
| Tocilizumab | 2 | NA | 2 | NA |
| **JAK-inhibitor** | 0 | NA | 4 | NA |
| Baricitinib | 0 | NA | 0 | NA |
| Tofacitinib | 0 | NA | 4 | NA |

CI: confidence interval; Disc.: discontinuation; esr: erythrocyte sedimentation rate; HR: hazard radio; inh.: inhibitor; JAK: janus kinase; TNF: tumor necrosis factor alpha;

Results in red color are significant results.

The following supplementary tables show results of comparative effectiveness analyses in clusters grouped together to make up group 4 (i.e. clusters of mainly seropositive patients with high RA disease burden and long RA disease duration, and with a tendency towards a higher proportion of women). The majority of clusters suggest a high risk of non-response with golimumab.

**Supplementary Table.** Comparative effectiveness analyses in the first cluster of group 4 (S5 Table)

|  | **Treatment disc. due to non-response** | **Crude HR**  **(95% CI)** | **20% DAS28-esr reduction** | **Crude HR**  **(95% CI)** |
| --- | --- | --- | --- | --- |
| **TNF-inhibitor** | 96 | Ref 1.00 | 71 | Ref 1.00 |
| Adalimumab | 34 | Ref 1.00 | 20 | Ref 1.00 |
| Certolizumab | 3 | NA | 0 | NA |
| Etanercept | 30 | 0.82 (0.50-1.33) | 31 | 1.57 (0.90-2.76) |
| Golimumab | 10 | 1.97 (0.98-3.99) | 5 | 1.93 (0.73-5.15) |
| Infliximab | 19 | 0.80 (0.46-1.40) | 15 | 1.29 (0.66-2.52) |
| **Non-TNF-inh.** | 12 | 0.97 (0.53-1.76) | 10 | 1.11 (0.57-2.15) |
| Abatacept | 5 | 1.11 (0.44-2.85) | 4 | NA |
| Rituximab | 2 | NA | 2 | NA |
| Tocilizumab | 5 | 1.06 (0.42-2.72) | 4 | NA |
| **JAK-inhibitor** | 3 | NA | 4 | NA |
| Baricitinib | 0 | NA | 2 | NA |
| Tofacitinib | 3 | NA | 2 | NA |

CI: confidence interval; Disc.: discontinuation; esr: erythrocyte sedimentation rate; HR: hazard radio; inh.: inhibitor; JAK: janus kinase; TNF: tumor necrosis factor alpha;

Results in red color are significant results.

**Supplementary Table.** Comparative effectiveness analyses in the second cluster of group 4 (S5 Table)

|  | **Treatment disc. due to non-response** | **Crude HR**  **(95% CI)** | **20% DAS28-esr reduction** | **Crude HR**  **(95% CI)** |
| --- | --- | --- | --- | --- |
| **TNF-inhibitor** | 63 | Ref 1.00 | 57 | Ref 1.00 |
| Adalimumab | 17 | Ref 1.00 | 17 | Ref 1.00 |
| Certolizumab | 0 | NA | 1 | NA |
| Etanercept | 21 | 0.92 (0.49-1.75) | 25 | 1.11 (0.60-2.06) |
| Golimumab | 9 | 4.54 (2.03-10.2) | 3 | NA |
| Infliximab | 16 | 1.13 (0.57-2.24) | 11 | 0.92 (0.43-1.97) |
| **Non-TNF-inh.** | 6 | 1.05 (0.45-2.42) | 3 | 0.61 (0.19-1.94) |
| Abatacept | 3 | NA | 1 | NA |
| Rituximab | 2 | NA | 1 | NA |
| Tocilizumab | 1 | NA | 1 | NA |
| **JAK-inhibitor** | 1 | NA | 4 | NA |
| Baricitinib | 0 | NA | 2 | NA |
| Tofacitinib | 1 | NA | 2 | NA |

CI: confidence interval; Disc.: discontinuation; esr: erythrocyte sedimentation rate; HR: hazard radio; inh.: inhibitor; JAK: janus kinase; TNF: tumor necrosis factor alpha;

Results in red color are significant results.

**Supplementary Table.** Comparative effectiveness analyses in the third cluster of group 4 (S5 Table)

|  | **Treatment disc. due to non-response** | **Crude HR**  **(95% CI)** | **20% DAS28-esr reduction** | **Crude HR**  **(95% CI)** |
| --- | --- | --- | --- | --- |
| **TNF-inhibitor** | 64 | Ref 1.00 | 55 | Ref 1.00 |
| Adalimumab | 20 | Ref 1.00 | 18 | Ref 1.00 |
| Certolizumab | 0 | NA | 0 | NA |
| Etanercept | 24 | 0.88 (0.49-1.60) | 26 | 1.14 (0.62-2.10) |
| Golimumab | 8 | 2.45 (1.08-5.56) | 3 | NA |
| Infliximab | 12 | 0.73 (0.36-1.49) | 8 | 0.68 (0.29-1.60) |
| **Non-TNF-inh.** | 9 | 1.15 (0.57-2.32) | 5 | 0.77 (0.31-1.93) |
| Abatacept | 4 | NA | 1 | NA |
| Rituximab | 1 | NA | 1 | NA |
| Tocilizumab | 4 | NA | 3 | NA |
| **JAK-inhibitor** | 1 | NA | 3 | NA |
| Baricitinib | 0 | NA | 2 | NA |
| Tofacitinib | 1 | NA | 1 | NA |

CI: confidence interval; Disc.: discontinuation; esr: erythrocyte sedimentation rate; HR: hazard radio; inh.: inhibitor; JAK: janus kinase; TNF: tumor necrosis factor alpha;

Results in red color are significant results.

**Supplementary Table.** Comparative effectiveness analyses in the fourth cluster of group 4 (S5 Table)

|  | **Treatment disc. due to non-response** | **Crude HR**  **(95% CI)** | **20% DAS28-esr reduction** | **Crude HR**  **(95% CI)** |
| --- | --- | --- | --- | --- |
| **TNF-inhibitor** | 91 | Ref 1.00 | 69 | Ref 1.00 |
| Adalimumab | 29 | Ref 1.00 | 21 | Ref 1.00 |
| Certolizumab | 2 | NA | 3 | NA |
| Etanercept | 35 | 0.99 (0.60-1.62) | 29 | 1.19 (0.68-2.09) |
| Golimumab | 12 | 3.26 (1.66-6.39) | 3 | NA |
| Infliximab | 13 | 0.66 (0.34-1.27) | 13 | 1.05 (0.53-2.10) |
| **Non-TNF-inh.** | 10 | 0.72 (0.38-1.38) | 8 | 0.82 (0.40-1.71) |
| Abatacept | 4 | NA | 1 | NA |
| Rituximab | 1 | NA | 1 | NA |
| Tocilizumab | 5 | 1.11 (0.43-2.87) | 6 | 2.40 (0.97-5.96) |
| **JAK-inhibitor** | 2 | NA | 5 | 3.36 (1.35-8.33) |
| Baricitinib | 0 | NA | 2 | NA |
| Tofacitinib | 2 | NA | 3 | NA |

CI: confidence interval; Disc.: discontinuation; esr: erythrocyte sedimentation rate; HR: hazard radio; inh.: inhibitor; JAK: janus kinase; TNF: tumor necrosis factor alpha;

Results in red color are significant results.

**Supplementary Table.** Comparative effectiveness analyses in the fifth cluster of group 4 (S5 Table)

|  | **Treatment disc. due to non-response** | **Crude HR**  **(95% CI)** | **20% DAS28-esr reduction** | **Crude HR**  **(95% CI)** |
| --- | --- | --- | --- | --- |
| **TNF-inhibitor** | 47 | Ref 1.00 | 48 | Ref 1.00 |
| Adalimumab | 15 | Ref 1.00 | 14 | Ref 1.00 |
| Certolizumab | 1 | NA | 1 | NA |
| Etanercept | 16 | 0.87 (0.43-1.76) | 22 | 1.39 (0.71-2.72) |
| Golimumab | 1 | NA | 0 | NA |
| Infliximab | 14 | 1.21 (0.59-2.51) | 11 | 1.28 (0.58-2.81) |
| **Non-TNF-inh.** | 4 | NA | 1 | NA |
| Abatacept | 3 | NA | 1 | NA |
| Rituximab | 0 | NA | 0 | NA |
| Tocilizumab | 1 | NA | 0 | NA |
| **JAK-inhibitor** | 1 | NA | 1 | NA |
| Baricitinib | 0 | NA | 1 | NA |
| Tofacitinib | 1 | NA | 1 | NA |

CI: confidence interval; Disc.: discontinuation; esr: erythrocyte sedimentation rate; HR: hazard radio; inh.: inhibitor; JAK: janus kinase; TNF: tumor necrosis factor alpha;

Results in red color are significant results.

**Supplementary Table.** Comparative effectiveness analyses in the sixth cluster of group 4 (S5 Table)

|  | **Treatment disc. due to non-response** | **Crude HR**  **(95% CI)** | **20% DAS28-esr reduction** | **Crude HR**  **(95% CI)** |
| --- | --- | --- | --- | --- |
| **TNF-inhibitor** | 96 | Ref 1.00 | 88 | Ref 1.00 |
| Adalimumab | 33 | Ref 1.00 | 27 | Ref 1.00 |
| Certolizumab | 2 | NA | 5 | 9.52 (3.66-24.8) |
| Etanercept | 34 | 0.92 (0.57-1.49) | 35 | 1.22 (0.74-2.00) |
| Golimumab | 8 | 3.68 (1.70-7.96) | 4 | NA |
| Infliximab | 19 | 0.92 (0.52-1.62) | 17 | 1.17 (0.64-2.20) |
| **Non-TNF-inh.** | 9 | 1.13 (0.57-2.24) | 10 | 1.57 (0.82-3.03) |
| Abatacept | 3 | NA | 3 | NA |
| Rituximab | 2 | NA | 3 | NA |
| Tocilizumab | 4 | NA | 4 | NA |
| **JAK-inhibitor** | 2 | NA | 3 | NA |
| Baricitinib | 0 | NA | 1 | NA |
| Tofacitinib | 2 | NA | 2 | NA |

CI: confidence interval; Disc.: discontinuation; esr: erythrocyte sedimentation rate; HR: hazard radio; inh.: inhibitor; JAK: janus kinase; TNF: tumor necrosis factor alpha;

Results in red color are significant results.

The following supplementary tables show results of comparative effectiveness analyses in clusters grouped together to make up group 5. The majority of clusters suggest a good response with golimumab and with tocilizumab.

**Supplementary Table.** Comparative effectiveness analyses in the first cluster of group 5 (S6 Table)

|  | **Treatment disc. due to non-response** | **Crude HR**  **(95% CI)** | **20% DAS28-esr reduction** | **Crude HR**  **(95% CI)** |
| --- | --- | --- | --- | --- |
| **TNF-inhibitor** | 123 | Ref 1.00 | 82 | Ref 1.00 |
| Adalimumab | 38 | Ref 1.00 | 22 | Ref 1.00 |
| Certolizumab | 0 | NA | 3 | NA |
| Etanercept | 46 | 1.06 (0.69-1.62) | 30 | 1.22 (0.70-2.10) |
| Golimumab | 20 | 3.16 (1.84-5.44) | 11 | 3.15 (1.53-6.50) |
| Infliximab | 19 | 0.82 (0.47-1.41) | 16 | 1.35 (0.71-2.60) |
| **Non-TNF-inh.** | 20 | 1.66 (1.04-2.66) | 12 | 1.61 (0.88-3.00) |
| Abatacept | 12 | 2.31 (1.21-4.43) | 3 | NA |
| Rituximab | 2 | NA | 3 | NA |
| Tocilizumab | 6 | 1.74 (0.74-4.12) | 6 | 3.56 (1.44-8.80) |
| **JAK-inhibitor** | 4 | NA | 8 | 4.11 (1.99-8.50) |
| Baricitinib | 0 | NA | 1 | NA |
| Tofacitinib | 4 | NA | 7 | 4.76 (2.03-11.1) |

CI: confidence interval; Disc.: discontinuation; esr: erythrocyte sedimentation rate; HR: hazard radio; inh.: inhibitor; JAK: janus kinase; TNF: tumor necrosis factor alpha;

Results in red color are significant results.

**Supplementary Table.** Comparative effectiveness analyses in the second cluster of group 5 (S6 Table)

|  | **Treatment disc. due to non-response** | **Crude HR**  **(95% CI)** | **20% DAS28-esr reduction** | **Crude HR**  **(95% CI)** |
| --- | --- | --- | --- | --- |
| **TNF-inhibitor** | 89 | Ref 1.00 | 55 | Ref 1.00 |
| Adalimumab | 32 | Ref 1.00 | 16 | Ref 1.00 |
| Certolizumab | 2 | NA | 2 | NA |
| Etanercept | 29 | 0.93 (0.56-1.53) | 17 | 1.17 (0.59-2.30) |
| Golimumab | 12 | 2.29 (1.18-4.45) | 7 | 2.83 (1.16-6.88) |
| Infliximab | 14 | 0.78 (0.42-1.47) | 13 | 1.76 (0.85-3.70) |
| **Non-TNF-inh.** | 18 | 1.64 (0.99-2.72) | 13 | 2.11 (1.15-3.86) |
| Abatacept | 8 | 1.64 (0.75-3.55) | 2 | NA |
| Rituximab | 2 | NA | 2 | NA |
| Tocilizumab | 8 | 2.01 (0.93-4.36) | 9 | 5.49 (2.42-12.4) |
| **JAK-inhibitor** | 4 | NA | 3 | NA |
| Baricitinib | 0 | NA | 1 | NA |
| Tofacitinib | 4 | NA | 2 | NA |

CI: confidence interval; Disc.: discontinuation; esr: erythrocyte sedimentation rate; HR: hazard radio; inh.: inhibitor; JAK: janus kinase; TNF: tumor necrosis factor alpha;

Results in red color are significant results.

**Supplementary Table.** Comparative effectiveness analyses in the third cluster of group 5 (S6 Table)

|  | **Treatment disc. due to non-response** | **Crude HR**  **(95% CI)** | **20% DAS28-esr reduction** | **Crude HR**  **(95% CI)** |
| --- | --- | --- | --- | --- |
| **TNF-inhibitor** | 48 | Ref 1.00 | 33 | Ref 1.00 |
| Adalimumab | 14 | Ref 1.00 | 7 | Ref 1.00 |
| Certolizumab | 3 | NA | 3 | NA |
| Etanercept | 13 | 0.86 (0.40-1.83) | 10 | 1.35 (0.51-3.54) |
| Golimumab | 6 | 2.48 (0.95-6.45) | 4 | NA |
| Infliximab | 12 | 1.56 (0.72-3.38) | 9 | 2.66 (0.99-7.15) |
| **Non-TNF-inh.** | 11 | 1.96 (1.02-3.77) | 7 | 2.04 (0.90-4.60) |
| Abatacept | 3 | NA | 2 | NA |
| Rituximab | 3 | NA | 0 | NA |
| Tocilizumab | 5 | 2.81 (1.01-7.81) | 5 | 6.43 (2.04-20.33) |
| **JAK-inhibitor** | 3 | NA | 2 | NA |
| Baricitinib | 0 | NA | 1 | NA |
| Tofacitinib | 3 | NA | 1 | NA |

CI: confidence interval; Disc.: discontinuation; esr: erythrocyte sedimentation rate; HR: hazard radio; inh.: inhibitor; JAK: janus kinase; TNF: tumor necrosis factor alpha;

Results in red color are significant results.

**Supplementary Table.** Comparative effectiveness analyses in the fourth cluster of group 5 (S6 Table)

|  | **Treatment disc. due to non-response** | **Crude HR**  **(95% CI)** | **20% DAS28-esr reduction** | **Crude HR**  **(95% CI)** |
| --- | --- | --- | --- | --- |
| **TNF-inhibitor** | 72 | Ref 1.00 | 49 | Ref 1.00 |
| Adalimumab | 25 | Ref 1.00 | 15 | Ref 1.00 |
| Certolizumab | 4 | NA | 3 | NA |
| Etanercept | 21 | 0.80 (0.45-1.44) | 16 | 1.04 (0.51-2.10) |
| Golimumab | 6 | 1.21 (0.50-2.94) | 7 | 2.74 (1.12-6.70) |
| Infliximab | 16 | 1.14 (0.61-2.13) | 8 | 1.11 (0.47-2.62) |
| **Non-TNF-inh.** | 15 | 1.49 (0.85-2.59) | 8 | 1.30 (0.62-2.75) |
| Abatacept | 5 | 1.10 (0.42-2.88) | 4 | NA |
| Rituximab | 3 | NA | 0 | NA |
| Tocilizumab | 7 | 1.99 (0.86-4.61) | 4 | NA |
| **JAK-inhibitor** | 3 | NA | 1 | NA |
| Baricitinib | 0 | NA | 1 | NA |
| Tofacitinib | 3 | NA | 0 | NA |

CI: confidence interval; Disc.: discontinuation; esr: erythrocyte sedimentation rate; HR: hazard radio; inh.: inhibitor; JAK: janus kinase; TNF: tumor necrosis factor alpha;

Results in red color are significant results.

**Supplementary Table.** Comparative effectiveness analyses in the fifth cluster of group 5 (S6 Table)

|  | **Treatment disc. due to non-response** | **Crude HR**  **(95% CI)** | **20% DAS28-esr reduction** | **Crude HR**  **(95% CI)** |
| --- | --- | --- | --- | --- |
| **TNF-inhibitor** | 100 | Ref 1.00 | 66 | Ref 1.00 |
| Adalimumab | 28 | Ref 1.00 | 20 | Ref 1.00 |
| Certolizumab | 5 | 1.80 (0.93-3.46) | 5 | 3.78 (1.42-10.08) |
| Etanercept | 37 | 1.07 (0.80-1.43) | 21 | 0.97 (0.53-1.79) |
| Golimumab | 15 | 1.56 (1.03-2.39) | 10 | 2.56 (1.20-5.47) |
| Infliximab | 15 | 1.02 (0.71-1.47) | 10 | 1.04 (0.49-2.23) |
| **Non-TNF-inh.** | 20 | 1.31 (0.98-1.76) | 14 | 1.53 (0.86-2.70) |
| Abatacept | 6 | 1.18 (0.72-1.92) | 5 | 1.42 (0.53-3.79) |
| Rituximab | 3 | NA | 0 | NA |
| Tocilizumab | 11 | 2.36 (1.17-4.74) | 9 | 3.39 (1.54-7.47) |
| **JAK-inhibitor** | 4 | NA | 5 | 4.12 (1.65-10.26) |
| Baricitinib | 0 | NA | 3 | NA |
| Tofacitinib | 4 | NA | 2 | NA |

CI: confidence interval; Disc.: discontinuation; esr: erythrocyte sedimentation rate; HR: hazard radio; inh.: inhibitor; JAK: janus kinase; TNF: tumor necrosis factor alpha;

Results in red color are significant results.
