# Supplementary figures and images for: Patient groups in Rheumatoid arthritis identified by deep learning respond differently to biologic or targeted synthetic DMARDs
Source: PLoS Comput Biol. 2023 Jun 2;19(6):e1011073. doi: 10.1371/journal.pcbi.1011073 (PMC10266686; doi:10.1371/journal.pcbi.1011073)

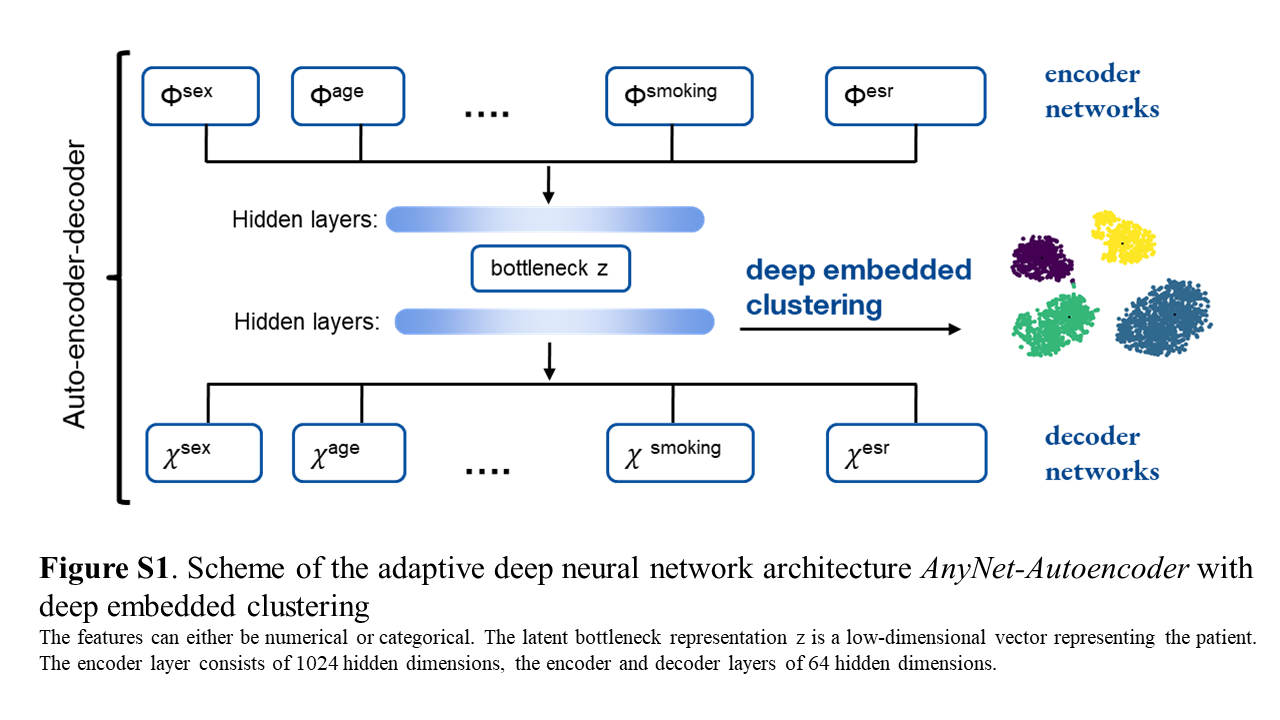

Supplement: S1 Fig — (TIF) [file pcbi.1011073.s005.tif]

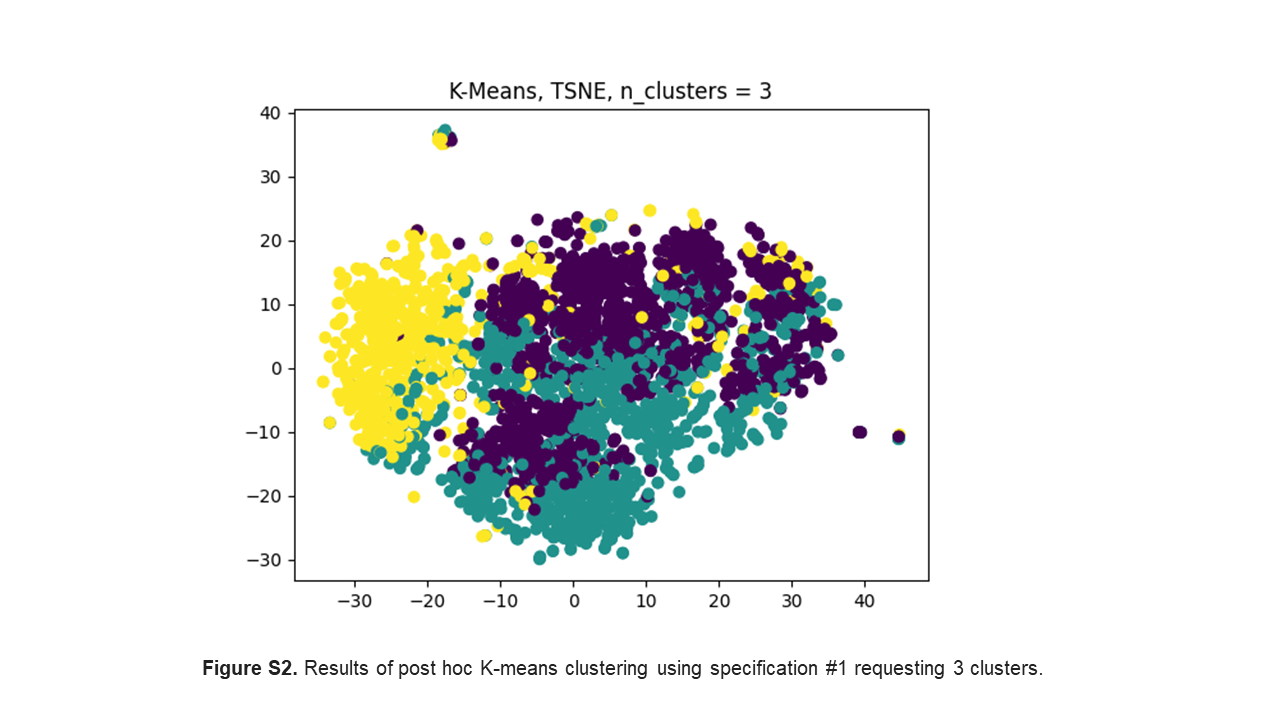

Supplement: S2 Fig — (TIF) [file pcbi.1011073.s006.tif]

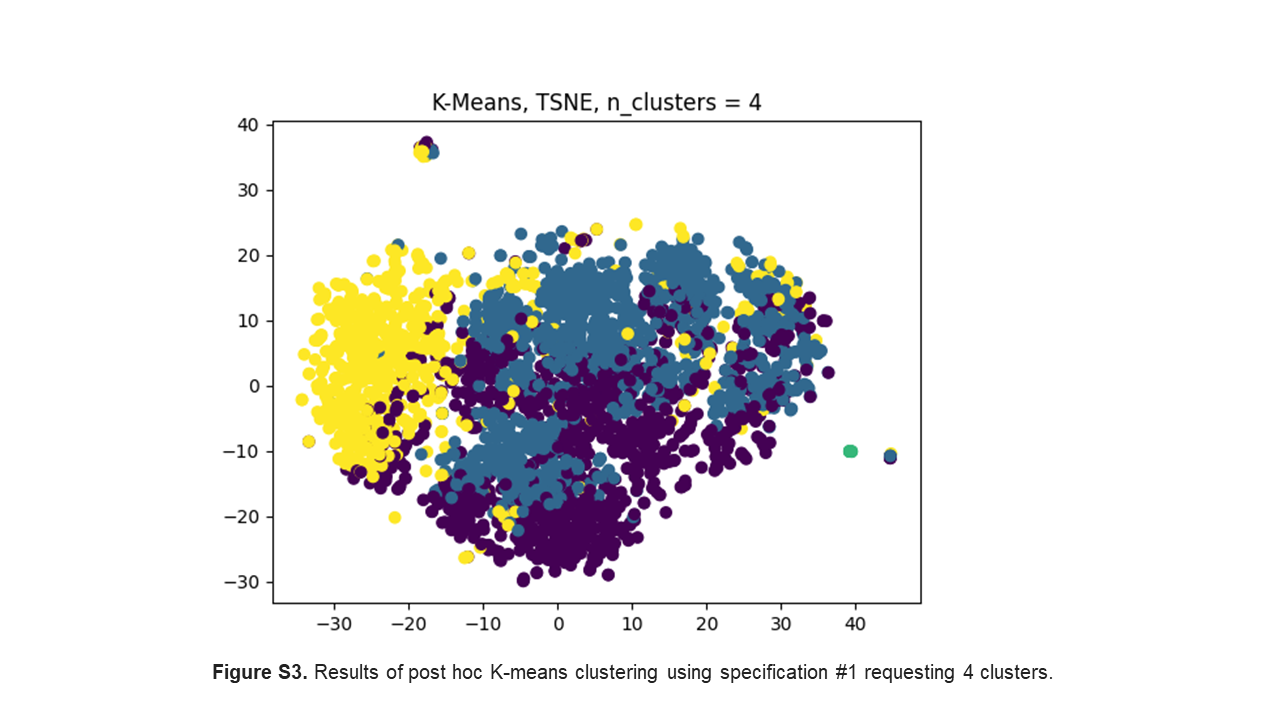

Supplement: S3 Fig — (TIF) [file pcbi.1011073.s007.tif]

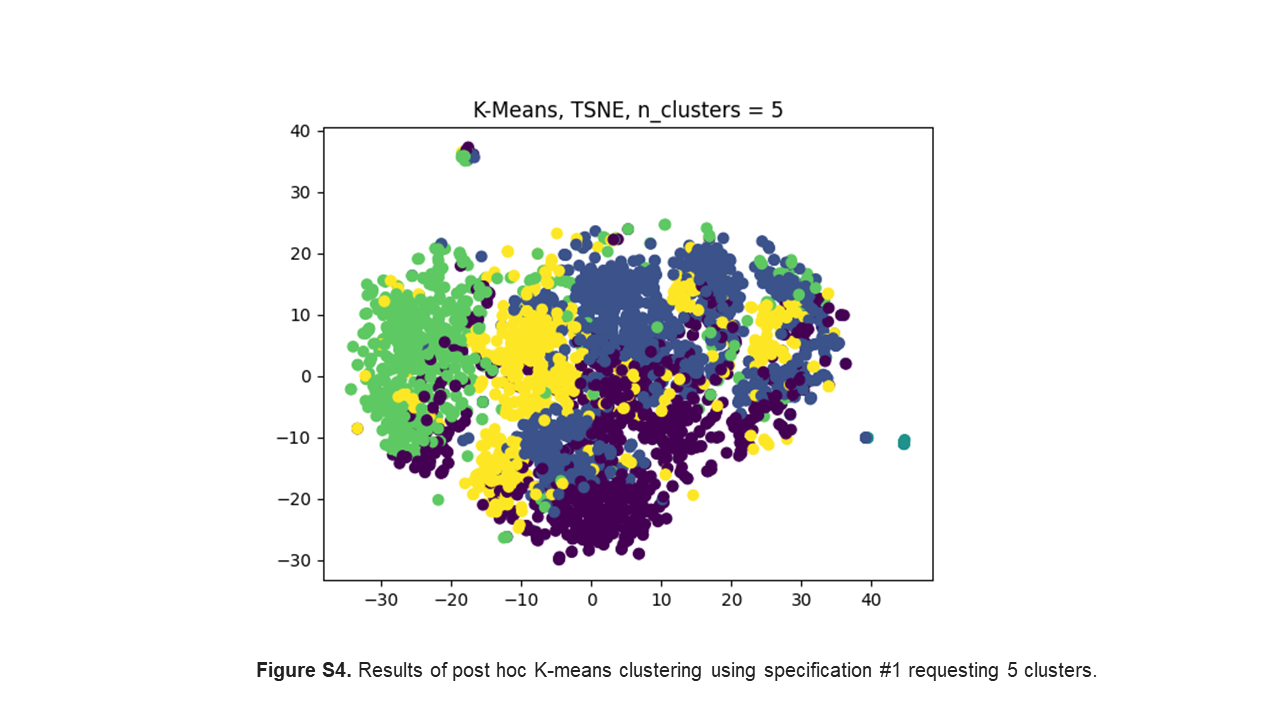

Supplement: S4 Fig — (TIF) [file pcbi.1011073.s008.tif]

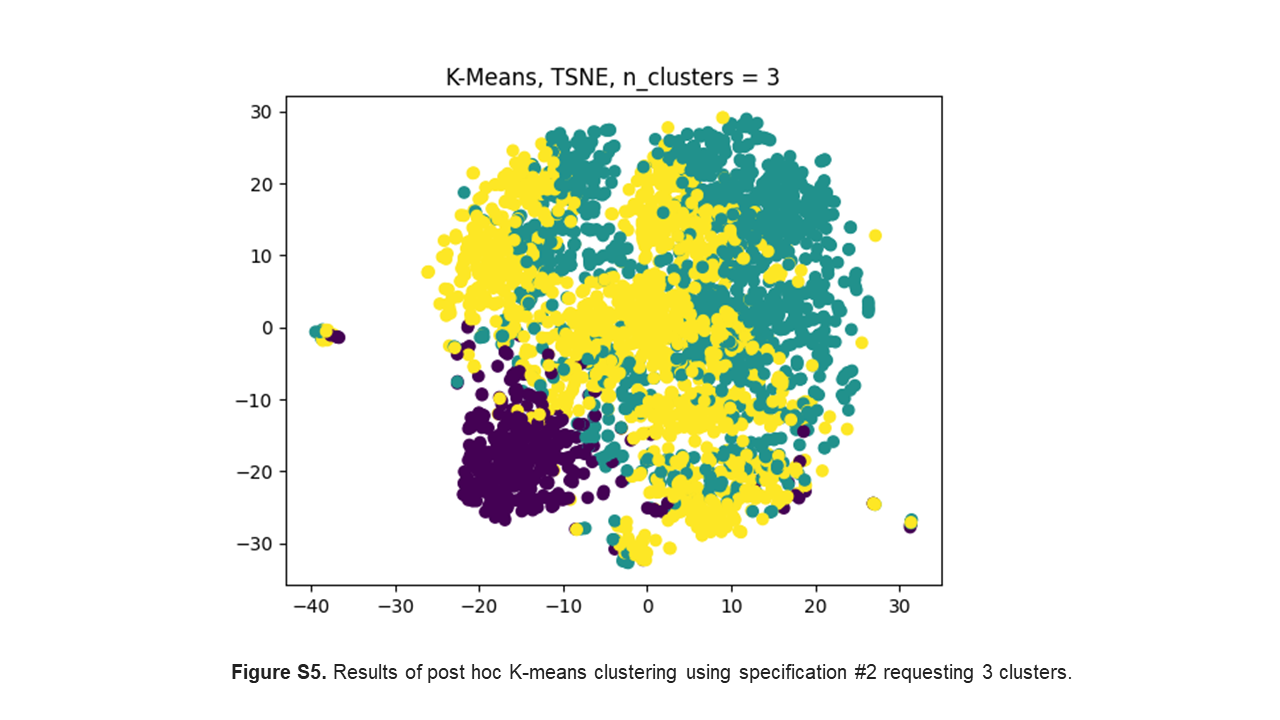

Supplement: S5 Fig — (TIF) [file pcbi.1011073.s009.tif]

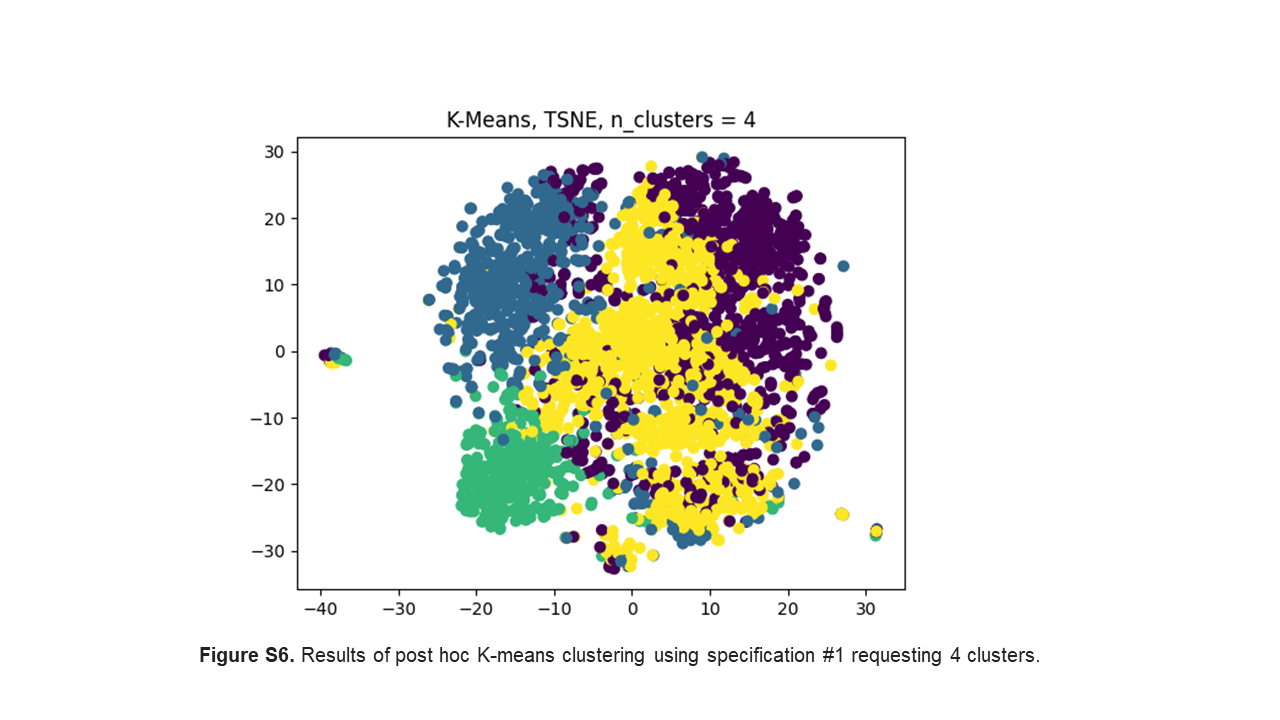

Supplement: S6 Fig — (TIF) [file pcbi.1011073.s010.tif]

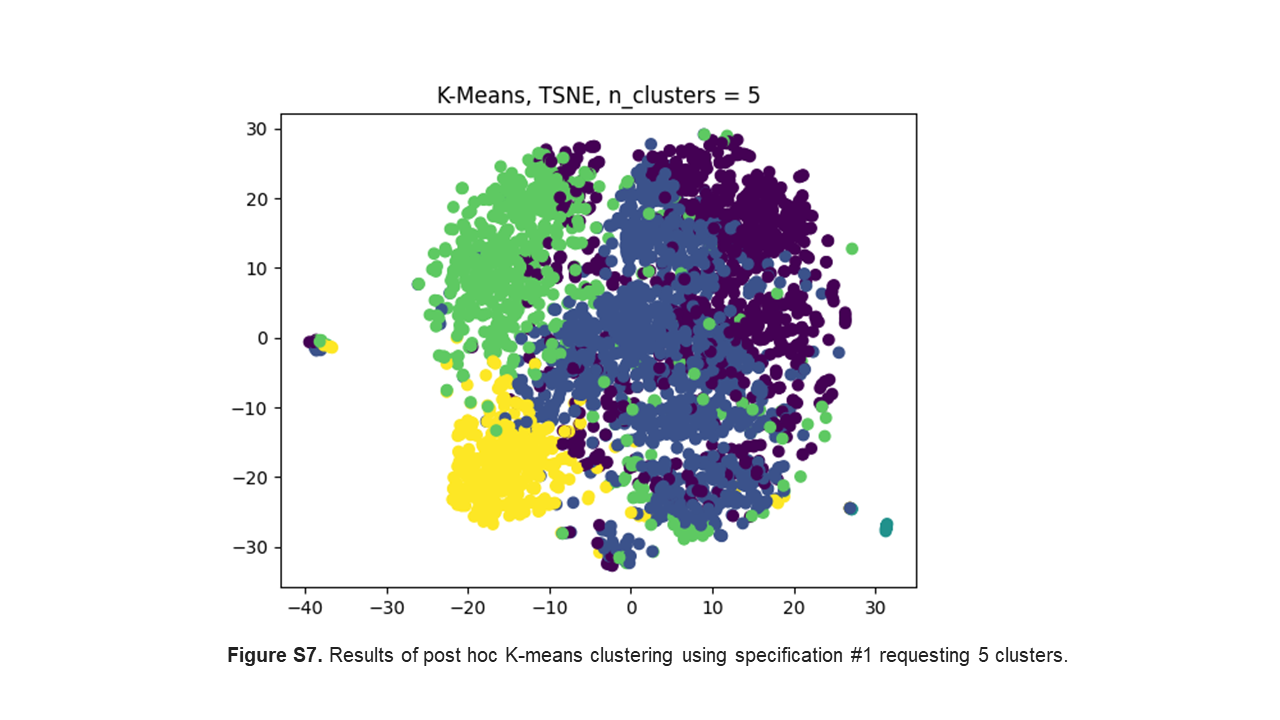

Supplement: S7 Fig — (TIF) [file pcbi.1011073.s011.tif]

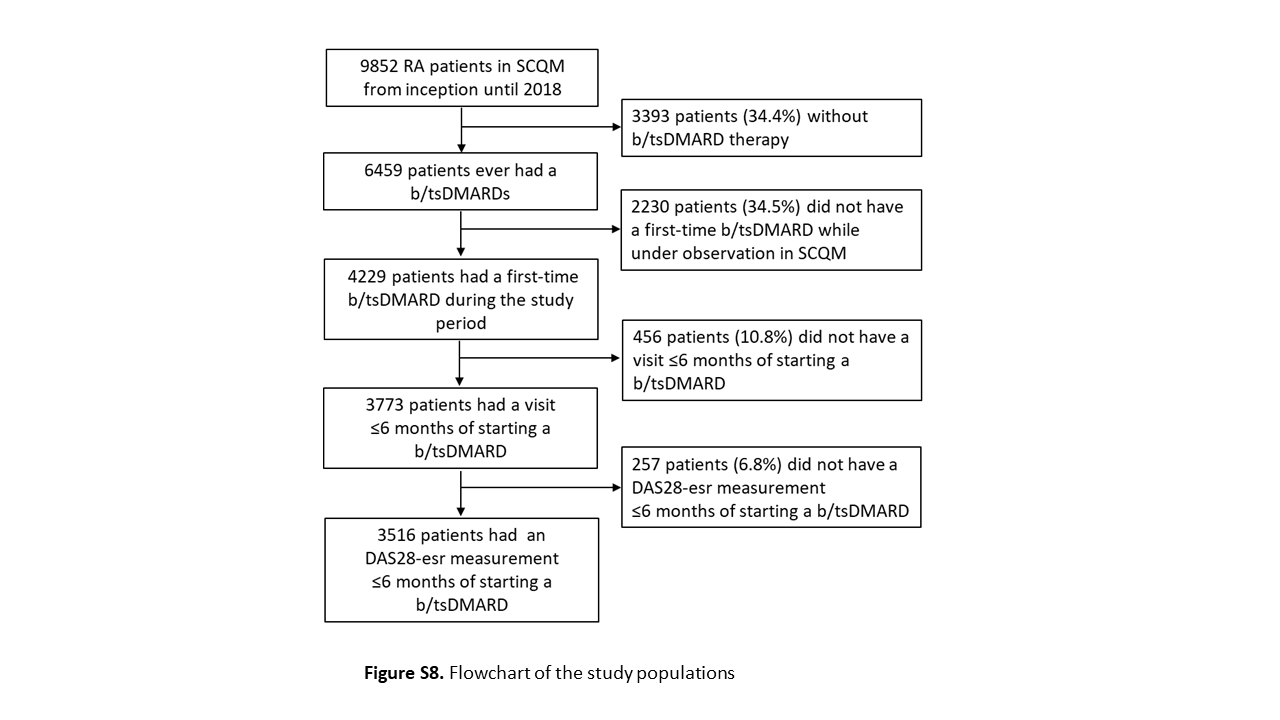

Supplement: S8 Fig — (TIF) [file pcbi.1011073.s012.tif]
