# Supplementary material for: Patient groups in Rheumatoid arthritis identified by deep learning respond differently to biologic or targeted synthetic DMARDs
Source: PLoS Comput Biol. 2023 Jun 2;19(6):e1011073. doi: 10.1371/journal.pcbi.1011073 (PMC10266686; doi:10.1371/journal.pcbi.1011073)
Supplement: S1 Table — (DOC) [file pcbi.1011073.s013.doc]

**Supplementary Table S1** Features used for clustering at cohort entry including variable type, missingness, lookback window, and clustering run

| Feature | type and amount of missingness (if applicable) | Lookback window | clustering runs # ^f^ |
| --- | --- | --- | --- |
| Age | continuous | ever before | 1 and 2 |
| Sex | binary: Male / Female | ever before | 1 and 2 |
| height | continuous | ever before | 1 and 2 |
| weight | continuous | ever before | 1 and 2 |
| BMI | continuous | ever before | 1 and 2 |
| Cycling, walking activity level ^a^ | None / low / moderate / high / 12.6% missing | ever before | 1 and 2 |
| Power sports activity level ^b^ | None / low / moderate / high / 13.3% missing | ever before | 1 and 2 |
| Smoking | Current / Former / Never smoker / 53.2% missing | ever before | 1 and 2 |
| numbers of years smoking (among current smokers) | continuous | ever before | 1 and 2 |
| smoking level among current smokers | >1 package per day / ≤1 package per day | ever before | 1 and 2 |
| RA duration ^c^ | continuous / 2.2% missing | ever before | 1 and 2 |
| Family history of rheumatic diseases ^d^ | Yes/ No / 36.7% missing | ever before | 1 and 2 |
| Rheumatoid factor titer | continuous / 81.0% missing | ever before | 1 and 2 |
| Rheumatoid factor | Yes/ No / 4.8% missing | ever before | 1 and 2 |
| ACPA titer | continuous / 79.6% missing | ever before | 1 and 2 |
| ACPA | Yes/ No / 31.0% missing | ever before | 1 and 2 |
| esr | continuous | 6 months | 1 and 2 |
| number of tender joints | continuous | 6 months | 1 and 2 |
| number of swollen joints | continuous | 6 months | 1 and 2 |
| crp | continuous / 57.1% missing | 6 months | 1 and 2 |
| activity of rheumatic disease | continuous / 13.4% missing | 6 months | 1 and 2 |
| morning stiffness | none / <30 min / 30 min - 1 hour / 1-2 hours / 2-4 hours / >4 hours / all day / 13.2% missing | 6 months | 1 and 2 |
| pain level VAS | continuous / 12.8% missing | 6 months | 1 and 2 |
| HAQ score | continuous / 13.8% missing | 6 months | 1 and 2 |
| EuroQoL score | continuous / 68.4% missing | 6 months | 1 and 2 |
| SF 12 mental component score | continuous / 23.4% missing | 6 months | 1 and 2 |
| SF 12 physical component score | continuous / 23.4% missing | 6 months | 1 and 2 |
| MTX use | Yes/ No | at cohort entry | 1 and 2 |
| duration of MTX use until cohort entry | continuous | at cohort entry | 1 and 2 |
| Leflunomid use | Yes/ No | at cohort entry | 1 and 2 |
| duration of leflunomid use until cohort entry | continuous | at cohort entry | 1 and 2 |
| Sulfosalazin use | Yes/ No | at cohort entry | 1 and 2 |
| duration of sulfosalazin use until cohort entry | continuous | at cohort entry | 1 and 2 |
| Chlorochine use | Yes/ No | at cohort entry | 1 and 2 |
| duration of chlorochine use until cohort entry | continuous | at cohort entry | 1 and 2 |
| Azathioprin use | Yes/ No | at cohort entry | 1 and 2 |
| duration of azathioprin use until cohort entry | continuous | at cohort entry | 1 and 2 |
| Cyclosporine use | Yes/ No | at cohort entry | 1 and 2 |
| duration of cyclosporine use | continuous | at cohort entry | 1 and 2 |
| Systemic  prednisone use | Yes/ No | at cohort entry | 1 and 2 |
| duration of systemic prednisone use | continuous | at cohort entry | 1 and 2 |
| sustained release systemic  prednisone use | Yes/ No | at cohort entry | 1 and 2 |
| duration of sustained release systemic prednisone use | continuous | at cohort entry | 1 and 2 |
| time elapsed since last measurement of esr ^e^ | continuous | 6 months | 2 only |
| time elapsed since last measurement of number of tender joints ^e^ | continuous | 6 months | 2 only |
| time elapsed since last measurement of number of swollen joints ^e^ | continuous | 6 months | 2 only |
| time elapsed since last measurement of morning stiffness ^e^ | continuous | 6 months | 2 only |
| time elapsed since last measurement of number of pain level VAS ^e^ | continuous | 6 months | 2 only |
| time elapsed since last measurement of number of HAQ score ^e^ | continuous | 6 months | 2 only |
| time elapsed since last measurement of number of EuroQoL score ^e^ | continuous | 6 months | 2 only |
| time elapsed since last measurement of number of SF 12 mental component score ^e^ | continuous | 6 months | 2 only |
| time elapsed since last measurement of number of SF 12 physical component score ^e^ | continuous | 6 months | 2 only |

ACPA: Anti-citrullinated protein antibodies; BMI: body mass index; CRP: C-reactive protein; DAS: disease activity score; DMARD: disease modifying anti-rheumatic drug, ESR: erythrocyte sedimentation rate; EuroQoL: a standardized instrument for measuring generic health status (EQ-5D), HAQ: health assessment questionnaire; RA: rheumatoid arthritis;  SF: Short form (health survey); VAS (visual analog scale)

^a^ low: <30 min daily walking / cycling, Moderate: 30-60 min daily walking / cycling, high: ≥60 min daily walking / cycling

^b^ low : <60 min power sports per week, Moderate: 1-2 h power sports per week, high:  ≥2 h power sports per week

^c^ RA duration assessed from diagnosis until cohort entry, if diagnosis date not available assessed RA duration from first symptoms minus 1 year

^d^ family history of rheumatic diseases includes rheumatoid arthritis, ankylosing spondylitis, psoriasis, psoriatic arthritis, chronic inflammatory bowel disease, and other spondyloarthropathies (e.g. reactive arthritis)

^e^ the use of these features allowed to account for the recency of the clinical measurement

^f^ input features for clustering runs #1 do not include those to account for recency of clinical measurements, however, input features for clustering runs #2 do
