# Supplementary material for: Patient groups in Rheumatoid arthritis identified by deep learning respond differently to biologic or targeted synthetic DMARDs
Source: PLoS Comput Biol. 2023 Jun 2;19(6):e1011073. doi: 10.1371/journal.pcbi.1011073 (PMC10266686; doi:10.1371/journal.pcbi.1011073)
Supplement: S2 Table — (DOC) [file pcbi.1011073.s014.doc]

**S2 Table.** Clusters with high use of conventional synthetic DMARDs and prednisone

|  | **Cluster**  **(n=1026)** | **Cluster**  **(n=758)** | **Cluster**  **(n=663)** | **Cluster**  **(n=1111)** | **Cluster**  **(n=362)** |
| --- | --- | --- | --- | --- | --- |
| **Mean age (SD) [years]** | 55.1 (13.8) | 56.4 (13.5) | 55.6 (13.7) | 55.9 (13.3) | 61 (10.8) |
| **Women (%)** | 802 (78.2%) | 587 (77.4%) | 569 (85.8%) | 823 (74.1%) | 251 (69.3%) |
| **Men (%)** | 224 (21.8%) | 171 (22.6%) | 94 (14.2%) | 288 (25.9%) | 111 (30.7%) |
| **Median RA duration (IQR) [% missing]** | 5.3 (2.1-12.4) [1.9%missing] | 6.6 (2.6-14.4) [2.1%missing] | 6 (2.3-13.7) [1.8%missing] | 5.1 (2.2-11.6) [1.4%missing] | 12 (5.1-20.6) [1.4%missing] |
| **Median Rheumatoid factor titer (IQR) [% missing]** | 89 (39-209) [63.6%missing] | 94 (40-217) [57.7%missing] | 80 (38.5-181.5) [61.4%missing] | 94.5 (40-207) [41.9%missing] | 100 (42-220) [72.9%missing] |
| **Rheumatoid factor negative (%)** | 285 (27.8%) | 135 (17.8%) | 132 (19.9%) | 2 (0.2%) | 160 (44.2%) |
| **Rheumatoid factor positive (%)** | 704 (68.6%) | 593 (78.2%) | 507 (76.5%) | 1050 (94.5%) | 183 (50.6%) |
| **Missing information** | 37 (3.6%) | 30 (4%) | 24 (3.6%) | 59 (5.3%) | 19 (5.3%) |
| **Median ACPA titer (IQR) [% missing]** | 191 (64-340) [63.3%missing] | 196 (82-340) [58.6%missing] | 200 (70-340) [61.8%missing] | 188 (61-340) [46.2%missing] | 186 (40-575) [64.1%missing] |
| **ACPA negative** | 286 (27.9%) | 170 (22.4%) | 161 (24.3%) | 155 (14%) | 116 (32%) |
| **ACPA positive** | 575 (56%) | 476 (62.8%) | 392 (59.1%) | 857 (77.1%) | 173 (47.8%) |
| **Missing information** | 165 (16.1%) | 112 (14.8%) | 110 (16.6%) | 99 (8.9%) | 73 (20.2%) |
| **No family history of rheumatic diseases** | 585 (57%) | 415 (54.8%) | 350 (52.8%) | 1098 (98.8%) | 186 (51.4%) |
| **Family history of rheumatic diseases** | 223 (21.7%) | 184 (24.3%) | 169 (25.5%) | 2 (0.2%) | 96 (26.5%) |
| **Missing information** | 218 (21.3%) | 159 (21%) | 144 (21.7%) | 11 (1%) | 80 (22.1%) |
| **Non-smoker** | 262 (25.5%) | 216 (28.5%) | 191 (28.8%) | 277 (24.9%) | 68 (18.8%) |
| **Current smoker** | 221 (21.5%) | 156 (20.6%) | 139 (21%) | 235 (21.2%) | 92 (25.4%) |
| **Mean no. of years smoking (SD)** | 28.1 (11.5) | 29.2 (11.1) | 28.4 (10.9) | 27.8 (11.2) | 36 (12.6) |
| **≤1 package per day** | 115 (11.2%) | 80 (10.6%) | 70 (10.6%) | 153 (13.8%) | 51 (14.1%) |
| **>1 package per day** | 21 (2.1%) | 20 (2.6%) | 13 (2%) | 18 (1.6%) | 5 (1.4%) |
| **Former smoker** | 197 (19.2%) | 158 (20.8%) | 121 (18.3%) | 225 (20.3%) | 90 (24.9%) |
| **Missing smoking** | 346 (33.7%) | 228 (30.1%) | 212 (32%) | 374 (33.7%) | 112 (30.9%) |
| **Mean BMI (SD) [% missing]** | 25.5 (5) [11.7%missing] | 25.3 (4.7) [10.7% missing] | 25.7 (5)  [9.8% missing] | 25.9 (5.1) [9.6%missing] | 26.8 (5.9) [8.8% missing] |
| **No low impact activity** | 90 (8.8%) | 70 (9.2%) | 60 (9.1%) | 97 (8.7%) | 47 (13%) |
| **Little low impact activity ^a^** | 262 (25.5%) | 193 (25.5%) | 164 (24.7%) | 276 (24.8%) | 102 (28.2%) |
| **Moderate low impact ^a^ activity** | 356 (34.7%) | 272 (35.9%) | 250 (37.7%) | 359 (32.3%) | 104 (28.7%) |
| **High low impact activity ^a^** | 207 (20.2%) | 152 (20.1%) | 143 (21.6%) | 227 (20.4%) | 62 (17.1%) |
| **Missing low impact activity** | 111 (10.8%) | 71 (9.4%) | 46 (6.9%) | 152 (13.7%) | 47 (13%) |
| **No power sports** | 404 (39.4%) | 309 (40.8%) | 264 (39.8%) | 456 (41%) | 48 (13.3%) |
| **Little power sports ^b^** | 173 (16.9%) | 129 (17%) | 116 (17.5%) | 166 (14.9%) | 196 (54.1%) |
| **Moderate power sports ^b^** | 198 (19.3%) | 149 (19.7%) | 137 (20.7%) | 210 (18.9%) | 53 (14.6%) |
| **High power sports ^b^** | 134 (13.1%) | 95 (12.5%) | 95 (14.3%) | 127 (11.4%) | 45 (12.4%) |
| **Missing information** | 117 (11.4%) | 76 (10%) | 51 (7.7%) | 152 (13.7%) | 20 (5.5%) |
| **No morning stiffness** | 297 (29%) | 229 (30.2%) | 216 (32.6%) | 323 (29.1%) | 64 (17.7%) |
| **Morning stiffness**  **<30 minutes** | 151 (14.7%) | 109 (14.4%) | 109 (16.4%) | 160 (14.4%) | 41 (11.3%) |
| **Morning stiffness**  **30 minutes –**  **1 hour** | 188 (18.3%) | 136 (17.9%) | 124 (18.7%) | 175 (15.8%) | 48 (13.3%) |
| **Morning stiffness**  **1-2 hours** | 106 (10.3%) | 72 (9.5%) | 59 (8.9%) | 126 (11.3%) | 41 (11.3%) |
| **Morning stiffness**  **2-4 hours** | 71 (6.9%) | 53 (7%) | 44 (6.6%) | 68 (6.1%) | 40 (11.1%) |
| **Morning stiffness**  **>4 hours** | 43 (4.2%) | 38 (5%) | 24 (3.6%) | 44 (4%) | 23 (6.4%) |
| **Morning stiffness all day** | 45 (4.4%) | 35 (4.6%) | 27 (4.1%) | 45 (4.1%) | 44 (12.2%) |
| **Missing information** | 125 (12.2%) | 229 (30.2%) | 60 (9.1%) | 170 (15.3%) | 61 (16.9%) |
| **DAS28-esr score (SD)** | 4.2 (1.4) | 4.1 (1.4) | 4.1 (1.4) | 4.3 (1.4) | 4.3 (1.4) |
| **EuroQol score (SD)**  **[% missing]** | 64.8 (20.6) [42.6%missing] | 64.2 (21.7)  [39.3% missing] | 66.4 (20.6)  [41.5% missing] | 63.8 (21) [43.8%missing] | 59.4 (21)  [47% missing] |
| **HAQ score (SD) [% missing]** | 0.9 (0.7) [12.8%missing] | 0.9 (0.7)  [12.4% missing] | 0.9 (0.7)  [9.8% missing] | 0.9 (0.7) [15.4%missing] | 1 (0.7)  [18% missing] |
| **Pain level today, scale 1-10 (SD) [% missing]** | 4.7 (2.8) [12.1%missing] | 4.7 (2.8) [11.2% missing] | 4.4 (2.7) [9% missing] | 4.6 (2.8) [15.4%missing] | 5.5 (2.7) [16.6% missing] |
| **Activity of rheumatic disease, scale 1-10 (SD) [% missing]** | 4.9 (2.6) [12.6%missing] | 4.9 (2.6) [11.7% missing] | 4.6 (2.6) [9.8% missing] | 4.9 (2.7) [16.1%missing] | 5.6 (2.5) [16.9% missing] |
| **SF12 physical component score (SD) [% missing]** | 36.1 (10) [21.4%missing] | 36.4 (10) [22.6% missing] | 37 (10.2) [19.5% missing] | 36.4 (10.3) [24.3%missing] | 34.2 (9.9) [28.7% missing] |
| **SF12 mental component score (SD) [% missing]** | 47 (11) [21.4%missing] | 47 (11.5) [22.6% missing] | 47.4 (11.2) [19.5% missing] | 46.7 (11.7) [24.3%missing] | 44.9 (12.3) [28.7% missing] |
| **Prednisone use (%)** | 566 (55.2%) | 426 (56.2%) | 380 (57.3%) | 522 (47%) | 183 (50.6%) |
| **Median use (IQR) [years]** | 1 (0.3-3.3) | 1.4 (0.4-5.1) | 1.2 (0.4-4.4) | 1.3 (0.5-3.9) | 4 (1.2-9.8) |
| **Methotrexate use (%)** | 836 (81.5%) | 639 (84.3%) | 547 (82.5%) | 869 (78.2%) | 274 (75.7%) |
| **Median use (IQR) [years]** | 1.7 (0.5-5) | 2.7 (0.8-6.5) | 2.1 (0.7-5.6) | 2 (0.7-5.2) | 4.1 (1.2-8.8) |
| **Leflunomid use (%)** | 341 (33.2%) | 260 (34.3%) | 222 (33.5%) | 347 (31.2%) | 149 (41.2%) |
| **Median use (IQR) [years]** | 1.2 (0.4-2.8) | 1.5 (0.6-3.7) | 1.5 (0.5-3.4) | 1.4 (0.6-3.6) | 4.4 (1.2-7.7) |
| **Sulfosalazin use (%)** | 250 (24.4%) | 205 (27%) | 170 (25.6%) | 218 (19.6%) | 90 (24.9%) |
| **Median use (IQR) [years]** | 2.3 (0.5-5.5) | 3 (0.7-7.1) | 2.5 (0.5-6.2) | 2.2 (0.8-6.3) | 5.6 (1.2-10.5) |

ACPA: Anti-citrullinated protein antibodies; BMI: body mass index; CRP: C-reactive protein; DAS: disease activity score; DMARD: disease modifying anti-rheumatic drug, ESR: erythrocyte sedimentation rate; EuroQoL: a standardized instrument for measuring generic health status (EQ-5D), HAQ: health assessment questionnaire; IQR: interquartile range, RA: rheumatoid arthritis;  SD: standard derivation, SF: Short form (health survey);

Features in red color were selected as parameters for stratified analysis.

^a^ low: <30 min daily walking / cycling, Moderate: 30-60 min daily walking / cycling, high: ≥60 min daily walking / cycling

^b^ low : <60 min power sports per week, Moderate: 1-2 h power sports per week, high:  ≥2 h power sports per week
