# Supplementary material for: Patient groups in Rheumatoid arthritis identified by deep learning respond differently to biologic or targeted synthetic DMARDs
Source: PLoS Comput Biol. 2023 Jun 2;19(6):e1011073. doi: 10.1371/journal.pcbi.1011073 (PMC10266686; doi:10.1371/journal.pcbi.1011073)
Supplement: S3 Table — (DOC) [file pcbi.1011073.s015.doc]

**S3 Table.** Clusters of men

|  | **Cluster**  **(n=712)** | **Cluster**  **(n=836)** | **Cluster**  **(n=726)** |
| --- | --- | --- | --- |
| **Mean age (SD) [years]** | 56 (12.8) | 56.3 (12.7) | 55.6 (12.8) |
| **Women (%)** | 7 (1%) | (0%) | 1 (0.1%) |
| **Men (%)** | 705 (99%) | 836 (100%) | 725 (99.9%) |
| **Median RA duration (IQR) [% missing]** | 4.9 (2.1-11.6)  [2%missing] | 4.9 (2-12.1)  [1.9%missing] | 4.6 (1.9-11.3) [2.2%missing] |
| **Median Rheumatoid factor titer (IQR) [% missing]** | 100 (44-271) [66.4%missing] | 100 (44-320) [67.1%missing] | 100 (41-275) [64.9%missing] |
| **Rheumatoid factor negative (%)** | 153 (21.5%) | 222 (26.6%) | 156 (21.5%) |
| **Rheumatoid factor positive (%)** | 527 (74%) | 582 (69.6%) | 541 (74.5%) |
| **Missing information** | 32 (4.5%) | 32 (3.8%) | 29 (4%) |
| **Median ACPA titer (IQR) [% missing]** | 190 (74-340) [67.6%missing] | 194 (79-340) [68.2%missing] | 195 (80-340) [67.1%missing] |
| **ACPA negative** | 147 (20.7%) | 211 (25.2%) | 168 (23.1%) |
| **ACPA positive** | 348 (48.9%) | 396 (47.4%) | 356 (49%) |
| **Missing information** | 217 (30.5%) | 229 (27.4%) | 202 (27.8%) |
| **No family history of rheumatic diseases** | 322 (45.2%) | 401 (48%) | 344 (47.4%) |
| **Family history of rheumatic diseases** | 131 (18.4%) | 151 (18.1%) | 129 (17.8%) |
| **Missing information** | 259 (36.4%) | 284 (34%) | 253 (34.9%) |
| **Non-smoker** | 65 (9.1%) | 90 (10.8%) | 73 (10.1%) |
| **Current smoker** | 206 (28.9%) | 241 (28.8%) | 211 (29.1%) |
| **Mean no. of years smoking (SD)** | 28.4 (11.9) | 28.2 (12) | 27.1 (11.4) |
| **≤1 package per day** | 108 (15.2%) | 124 (14.8%) | 110 (15.2%) |
| **>1 package per day** | 23 (3.2%) | 26 (3.1%) | 24 (3.3%) |
| **Former smoker** | 128 (18%) | 161 (19.3%) | 134 (18.5%) |
| **Missing smoking** | 313 (44%) | 344 (41.2%) | 308 (42.4%) |
| **Mean BMI (SD) [% missing]** | 26.4 (4.2) [7.3% missing] | 26.4 (4.1) [9.2% missing] | 26.4 (4.1) [9.4% missing] |
| **No low impact activity** | 100 (14%) | 120 (14.4%) | 103 (14.2%) |
| **Little low impact activity ^a^** | 177 (24.9%) | 199 (23.8%) | 173 (23.8%) |
| **Moderate low impact ^a^ activity** | 186 (26.1%) | 221 (26.4%) | 196 (27%) |
| **High low impact activity ^a^** | 155 (21.8%) | 188 (22.5%) | 161 (22.2%) |
| **Missing low impact activity** | 94 (13.2%) | 108 (12.9%) | 93 (12.8%) |
| **No power sports** | 300 (42.1%) | 345 (41.3%) | 289 (39.8%) |
| **Little power sports ^b^** | 98 (13.8%) | 116 (13.9%) | 100 (13.8%) |
| **Moderate power sports ^b^** | 120 (16.9%) | 143 (17.1%) | 128 (17.6%) |
| **High power sports ^b^** | 96 (13.5%) | 119 (14.2%) | 113 (15.6%) |
| **Missing information** | 98 (13.8%) | 113 (13.5%) | 96 (13.2%) |
| **No morning stiffness** | 202 (28.4%) | 245 (29.3%) | 224 (30.9%) |
| **Morning stiffness <30 minutes** | 93 (13.1%) | 114 (13.6%) | 105 (14.5%) |
| **Morning stiffness 30 minutes – 1 hour** | 135 (19%) | 149 (17.8%) | 128 (17.6%) |
| **Morning stiffness 1-2 hours** | 78 (11%) | 90 (10.8%) | 81 (11.2%) |
| **Morning stiffness 2-4 hours** | 51 (7.2%) | 58 (6.9%) | 48 (6.6%) |
| **Morning stiffness >4 hours** | 31 (4.4%) | 33 (4%) | 27 (3.7%) |
| **Morning stiffness all day** | 33 (4.6%) | 38 (4.6%) | 24 (3.3%) |
| **Missing information** | 89 (12.5%) | 109 (13%) | 89 (12.3%) |
| **DAS28-esr score (SD)** | 4.2 (1.5) | 4.1 (1.5) [0% missing] | 4.2 (1.5) |
| **EuroQol score (SD) [% missing]** | 63.2 (21.1)  [62.5% missing] | 64.1 (21.9)  [59.6% missing] | 64.6 (22) [60.9% missing] |
| **HAQ score (SD) [% missing]** | 0.9 (0.7) [13.1% missing] | 0.9 (0.7) [13.6% missing] | 0.9 (0.7) [12.8% missing] |
| **Pain level today, scale 1-10 (SD) [% missing]** | 4.7 (2.8) [11.9% missing] | 4.6 (2.8) [12.2% missing] | 4.6 (2.8) [11.6% missing] |
| **Activity of rheumatic disease, scale 1-10 (SD) [% missing]** | 4.9 (2.7) [12.4% missing] | 4.8 (2.7) [12.7% missing] | 4.8 (2.8) [12% missing] |
| **SF12 physical component score (SD) [% missing]** | 35.2 (9.8) [18.7% missing] | 35.4 (10) [19.7% missing] | 35.4 (10) [18.3% missing] |
| **SF12 mental component score (SD) [% missing]** | 45.7 (11.9)  [18.7% missing] | 45.7 (12) [19.7% missing] | 46.1 (11.9)  [18.3% missing] |
| **Prednison use (%)** | 295 (41.4%) | 360 (43.1%) | 303 (41.7%) |
| **Median use (IQR) [years]** | 1 (0.4-2.6) | 1.0 (0.4-2.7) | 1 (0.4-2.3) |
| **Methotrexate use (%)** | 454 (63.8%) | 548 (65.6%) | 468 (64.5%) |
| **Median use (IQR) [years]** | 1.5 (0.6-4) | 1.5 (0.5-4.0) | 1.3 (0.5-3.7) |
| **Leflunomid use (%)** | 167 (23.5%) | 199 (23.8%) | 163 (22.5%) |
| **Median use (IQR) [years]** | 1.1 (0.5-2.6) | 1.2 (0.6-2.6) | 1.1 (0.5-2.3) |
| **Sulfosalazin use (%)** | 105 (14.8%) | 131 (15.7%) | 104 (14.3%) |
| **Median use (IQR) [years]** | 2.1 (0.7-4.2) | 2.1 (0.7-4.4) | 2.3 (0.8-4.5) |

ACPA: Anti-citrullinated protein antibodies; BMI: body mass index; CRP: C-reactive protein; DAS: disease activity score; DMARD: disease modifying anti-rheumatic drug, ESR: erythrocyte sedimentation rate; EuroQoL: a standardized instrument for measuring generic health status (EQ-5D), HAQ: health assessment questionnaire; IQR: interquartile range, RA: rheumatoid arthritis; SD: standard derivation, SF: Short form (health survey);

Features in red color were selected as parameters for stratified analysis.

^a^ low: <30 min daily walking / cycling, Moderate: 30-60 min daily walking / cycling, high: ≥60 min daily walking / cycling

^b^ low : <60 min power sports per week, Moderate: 1-2 h power sports per week, high:  ≥2 h power sports per week
