# Supplementary material for: Patient groups in Rheumatoid arthritis identified by deep learning respond differently to biologic or targeted synthetic DMARDs
Source: PLoS Comput Biol. 2023 Jun 2;19(6):e1011073. doi: 10.1371/journal.pcbi.1011073 (PMC10266686; doi:10.1371/journal.pcbi.1011073)
Supplement: S4 Table — (DOC) [file pcbi.1011073.s016.doc]

**S4 Table.** Clusters of seronegative patients with a tendency towards low use of prednisone and a higher proportion of women

|  | Cluster  (n=799) | Cluster  (n=649) | Cluster  (n=933) | Cluster  (n=713) | Cluster  (n=613) |
| --- | --- | --- | --- | --- | --- |
| Mean age (SD) [years] | 53 (14.1) | 53.4 (14.2) | 53.4 (14) | 53.6 (14.2) | 52.6 (14.5) |
| Women (%) | 609 (76.2%) | 611 (94.1%) | 712 (76.3%) | 712 (99.9%) | 613 (100%) |
| Men (%) | 190 (23.8%) | 38 (5.9%) | 221 (23.7%) | 1 (0.1%) | (0%) |
| Median RA duration (IQR) [% missing] | 4.5 (1.8-10.7) [0.9% missing] | 4.8 (1.9-10.9) [0.9%missing] | 4.8 (1.9-10.9) [1%missing] | 4.9 (2-11.2) [1% missing] | 4.2 (1.8-9.6) [1%missing] |
| Median Rheumatoid factor titer (IQR) [% missing] | All missing | 36 (36-36) [99.8%missing] | All missing | All missing | All missing |
| Rheumatoid factor negative (%) | 798 (99.9%) | 645 (99.4%) | 931 (99.8%) | 711 (99.7%) | 611 (99.7%) |
| Rheumatoid factor positive (%) | (0%) | 2 (0.3%) | 1 (0.1%) | 1 (0.1%) | (0%) |
| Missing information | 1 (0.1%) | 2 (0.3%) | 1 (0.1%) | 1 (0.1%) | 2 (0.3%) |
| ACPA negative | 449 (56.2%) | 390 (60.1%) | 540 (57.9%) | 413 (57.9%) | 363 (59.2%) |
| ACPA positive | 126 (15.8%) | 101 (15.6%) | 154 (16.5%) | 127 (17.8%) | 97 (15.8%) |
| Missing information | 224 (28%) | 158 (24.4%) | 239 (25.6%) | 173 (24.3%) | 153 (25%) |
| No family history of rheumatic diseases | 408 (51.1%) | 343 (52.9%) | 487 (52.2%) | 374 (52.5%) | 318 (51.9%) |
| Family history of rheumatic diseases | 156 (19.5%) | 137 (21.1%) | 185 (19.8%) | 152 (21.3%) | 126 (20.6%) |
| Missing information | 235 (29.4%) | 169 (26%) | 261 (28%) | 187 (26.2%) | 169 (27.6%) |
| Non-smoker | 166 (20.8%) | 152 (23.4%) | 212 (22.7%) | 177 (24.8%) | 157 (25.6%) |
| Current smoker | 162 (20.3%) | 127 (19.6%) | 182 (19.5%) | 132 (18.5%) | 111 (18.1%) |
| Mean no. of years smoking (SD) | 23.9 (13.8) | 23.8 (14) | 24.1 (13.6) | 24 (13.5) | 22.2 (12.4) |
| ≤1 package per day | 92 (11.5%) | 76 (11.7%) | 100 (10.7%) | 77 (10.8%) | 68 (11.1%) |
| >1 package per day | 10 (1.3%) | 9 (1.4%) | 12 (1.3%) | 8 (1.1%) | 5 (0.8%) |
| Former smoker | 106 (13.3%) | 88 (13.6%) | 130 (13.9%) | 91 (12.8%) | 66 (10.8%) |
| Missing smoking | 365 (45.7%) | 282 (43.5%) | 409 (43.8%) | 313 (43.9%) | 279 (45.5%) |
| Mean BMI (SD) [% missing] | 26.2 (5.4) [10.8% missing] | 26 (5.6) [12.8% missing] | 26.1 (5.3) [11.7%missing] | 25.9 (5.6) [11.5% missing] | 25.9 (5.4) [11.7% missing] |
| No low impact activity | 103 (12.9%) | 79 (12.2%) | 114 (12.2%) | 81 (11.4%) | 82 (13.4%) |
| Little low impact activity ^a^ | 201 (25.2%) | 162 (25%) | 231 (24.8%) | 178 (25%) | 65 (10.6%) |
| Moderate low impact ^a^ activity | 248 (31%) | 210 (32.4%) | 292 (31.3%) | 237 (33.2%) | 148 (24.1%) |
| High low impact activity ^a^ | 142 (17.8%) | 107 (16.5%) | 176 (18.9%) | 121 (17%) | 209 (34.1%) |
| Missing low impact activity | 105 (13.1%) | 91 (14%) | 120 (12.9%) | 96 (13.5%) | 109 (17.8%) |
| No power sports | 344 (43.1%) | 290 (44.7%) | 386 (41.4%) | 300 (42.1%) | 248 (40.5%) |
| Little power sports ^b^ | 105 (13.1%) | 87 (13.4%) | 136 (14.6%) | 108 (15.2%) | 92 (15%) |
| Moderate power sports ^b^ | 138 (17.3%) | 104 (16%) | 160 (17.2%) | 120 (16.8%) | 107 (17.5%) |
| High power sports ^b^ | 98 (12.3%) | 69 (10.6%) | 119 (12.8%) | 80 (11.2%) | 75 (12.2%) |
| Missing information | 114 (14.3%) | 99 (15.3%) | 132 (14.2%) | 105 (14.7%) | 91 (14.9%) |
| No morning stiffness | 194 (24.3%) | 140 (21.6%) | 233 (25%) | 161 (22.6%) | 145 (23.7%) |
| Morning stiffness <30 minutes | 117 (14.6%) | 100 (15.4%) | 144 (15.4%) | 113 (15.9%) | 104 (17%) |
| Morning stiffness 30 minutes – 1 hour | 138 (17.3%) | 113 (17.4%) | 161 (17.3%) | 130 (18.2%) | 117 (19.1%) |
| Morning stiffness 1-2 hours | 89 (11.1%) | 80 (12.3%) | 99 (10.6%) | 84 (11.8%) | 72 (11.8%) |
| Morning stiffness 2-4 hours | 69 (8.6%) | 53 (8.2%) | 78 (8.4%) | 57 (8%) | 43 (7%) |
| Morning stiffness >4 hours | 27 (3.4%) | 23 (3.5%) | 29 (3.1%) | 22 (3.1%) | 18 (2.9%) |
| Morning stiffness all day | 51 (6.4%) | 43 (6.6%) | 56 (6%) | 44 (6.2%) | 28 (4.6%) |
| Missing information | 114 (14.3%) | 97 (15%) | 133 (14.3%) | 102 (14.3%) | 86 (14%) |
| DAS28-esr score (SD) | 4.2 (1.4) | 4.2 (1.3) | 4.1 (1.4) | 4.2 (1.3) | 4.2 (1.4) |
| HAQ score (SD) [% missing] | 1 (0.7) [14.3% missing] | 1 (0.7) [14.9% missing] | 1 (0.7) [14.3%missing] | 1 (0.7) [14.3% missing] | 1 (0.7) [13.7% missing] |
| Pain level today, scale 1-10 (SD) [% missing] | 5.1 (2.7) [14.3% missing] | 5.3 (2.7) [14.9% missing] | 5 (2.7) [14.1%missing] | 5.3 (2.7) [14.6% missing] | 5.2 (2.7) [14.2% missing] |
| Activity of rheumatic disease, scale 1-10 (SD) [% missing] | 5.2 (2.6) [14.5% missing] | 5.4 (2.6) [15.1% missing] | 5.2 (2.6) [14.7%missing] | 5.4 (2.6) [15% missing] | 5.3 (2.6) [14.7% missing] |
| SF12 physical component score (SD) [% missing] | 34.5 (9.8) [22.3% missing] | 33.7 (9.3) [23.4% missing] | 34.8 (9.8) [22.3%missing] | 34.1 (9.5) [22.7% missing] | 34.1 (9.5) [22% missing] |
| SF12 mental component score (SD) [% missing] | 44.8 (12) [22.3% missing] | 44.9 (12.1) [23.4% missing] | 45 (12) [22.3%missing] | 45.4 (11.9) [22.7% missing] | 45.4 (11.8) [22% missing] |
| Prednison use (%) | 292 (36.6%) | 229 (35.3%) | 367 (39.3%) | 268 (37.6%) | 225 (36.7%) |
| Median use (IQR) [years] | 0.9 (0.4-2) | 0.9 (0.4-2.2) | 1 (0.4-2.6) | 1 (0.4-2.9) | 0.9 (0.4-2) |
| Methotrexate use (%) | 506 (63.3%) | 410 (63.2%) | 612 (65.6%) | 465 (65.2%) | 399 (65.1%) |
| Median use (IQR) [years] | 1 (0.5-2.8) | 1.2 (0.5-3.3) | 1.1 (0.5-3.2) | 1.3 (0.5-3.6) | 1 (0.5-2.5) |
| Leflunomid use (%) | 196 (24.5%) | 166 (25.6%) | 242 (25.9%) | 193 (27.1%) | 153 (25%) |
| Median use (IQR) [years] | 1 (0.4-2) | 1 (0.4-2.2) | 1 (0.4-2.5) | 1 (0.4-2.4) | 0.7 (0.3-1.5) |
| Sulfosalazin use (%) | 141 (17.7%) | 123 (19%) | 178 (19.1%) | 143 (20.1%) | 119 (19.4%) |
| Median use (IQR) [years] | 1.2 (0.5-3.1) | 1.2 (0.6-4.8) | 1.2 (0.5-4) | 1.3 (0.6-4.8) | 1.2 (0.5-3) |

ACPA: Anti-citrullinated protein antibodies; BMI: body mass index; CRP: C-reactive protein; DAS: disease activity score; DMARD: disease modifying anti-rheumatic drug, ESR: erythrocyte sedimentation rate; EuroQoL: a standardized instrument for measuring generic health status (EQ-5D), HAQ: health assessment questionnaire; IQR: interquartile range, RA: rheumatoid arthritis; SD: standard derivation, SF: Short form (health survey);

Features in red color were selected as parameters for stratified analysis.

^a^ low: <30 min daily walking / cycling, Moderate: 30-60 min daily walking / cycling, high: ≥60 min daily walking / cycling

^b^ low : <60 min power sports per week, Moderate: 1-2 h power sports per week, high:  ≥2 h power sports per week
