# Supplementary material for: Patient groups in Rheumatoid arthritis identified by deep learning respond differently to biologic or targeted synthetic DMARDs
Source: PLoS Comput Biol. 2023 Jun 2;19(6):e1011073. doi: 10.1371/journal.pcbi.1011073 (PMC10266686; doi:10.1371/journal.pcbi.1011073)
Supplement: S5 Table — (DOC) [file pcbi.1011073.s017.doc]

**S5 Table.** Clusters of mainly seropositive patients with high RA disease burden and long RA disease duration, and with a tendency towards a higher proportion of women

|  | **Cluster**  **(n=1009)** | **Cluster**  **(n=817)** | **Cluster**  **(n=804)** | **Cluster**  **(n=958)** | **Cluster**  **(n=669)** | **Cluster**  **(n=1472)** |
| --- | --- | --- | --- | --- | --- | --- |
| **Mean age (SD) [years]** | 60.8 (11.2) | 59.4 (12.3) | 59.2 (12.3) | 56.8 (13.2) | 57.8 (13.1) | 56.4 (13.5) |
| **Women (%)** | 900 (89.2%) | 733 (89.7%) | 804 (100%) | 958 (100%) | 669 (100%) | 1144 (77.7%) |
| **Men (%)** | 109 (10.8%) | 84 (10.3%) | (0%) | (0%) | (0%) | 328 (22.3%) |
| **Median RA duration (IQR) [% missing]** | 12.2 (6.1-20.3)  [2.5% missing] | 11.7 (5.4-20) [3.3% missing] | 11.7 (5.2-20.8) [2.6% missing] | 8.3 (3.4-14.8) [2.6% missing] | 14.8 (8-23.6) [2.1% missing] | 8.6 (3.6-16.7) [3% missing] |
| **Rheumatoid factor negative (%)** | 136 (13.5%) | (0%) | 3 (0.4%) | (0%) | 6 (0.9%) | (0%) |
| **Rheumatoid factor positive (%)** | 814 (80.7%) | 758 (92.8%) | 746 (92.8%) | 902 (94.2%) | 626 (93.6%) | 1384 (94%) |
| **Missing information** | 59 (5.9%) | 59 (7.2%) | 55 (6.8%) | 56 (5.9%) | 37 (5.5%) | 88 (6%) |
| **ACPA negative** | 145 (14.4%) | 105 (12.9%) | 93 (11.6%) | 141 (14.7%) | 72 (10.8%) | 202 (13.7%) |
| **ACPA positive** | 513 (50.8%) | 375 (45.9%) | 409 (50.9%) | 512 (53.4%) | 319 (47.7%) | 645 (43.8%) |
| **Missing information** | 351 (34.8%) | 337 (41.3%) | 302 (37.6%) | 305 (31.8%) | 278 (41.6%) | 625 (42.5%) |
| **No family history of rheumatic diseases** | 410 (40.6%) | 241 (29.5%) | 279 (34.7%) | 383 (40%) | 141 (21.1%) | 4 (0.3%) |
| **Family history of rheumatic diseases** | 216 (21.4%) | 202 (24.7%) | 186 (23.1%) | 218 (22.8%) | 240 (35.9%) | 601 (40.8%) |
| **Missing information** | 383 (38%) | 374 (45.8%) | 339 (42.2%) | 357 (37.3%) | 288 (43.1%) | 867 (58.9%) |
| **Non-smoker** | 144 (14.3%) | 82 (10%) | 100 (12.4%) | 132 (13.8%) | 94 (14.1%) | 163 (11.1%) |
| **Current smoker** | 166 (16.5%) | 176 (21.5%) | 153 (19%) | 184 (19.2%) | 106 (15.8%) | 327 (22.2%) |
| **Mean no. of years smoking (SD)** | 31.8 (12.4) | 29.9 (12.1) | 29.2 (12) | 27.7 (11.5) | 27.4 (12.8) | 29.2 (11.6) |
| **≤1 package per day** | 106 (10.5%) | 106 (13%) | 97 (12.1%) | 112 (11.7%) | 62 (9.3%) | 161 (10.9%) |
| **>1 package per day** | 5 (0.5%) | 10 (1.2%) | 4 (0.5%) | 11 (1.2%) | 4 (0.6%) | 24 (1.6%) |
| **Former smoker** | 131 (13%) | 51 (6.2%) | 69 (8.6%) | 90 (9.4%) | 22 (3.3%) | 113 (7.7%) |
| **Missing smoking** | 568 (56.3%) | 508 (62.2%) | 482 (60%) | 552 (57.6%) | 447 (66.8%) | 869 (59%) |
| **Mean BMI (SD) [% missing]** | 26.5 (5.6) [6.6%missing] | 25.4 (5.2)  [7% missing] | 25.1 (5.2)  [6.7% missing] | 26.3 (5.4)  [8.8% missing] | 23.6 (4.1)  [5.4% missing] | 24.9 (4.7) [5.6%missing] |
| **No low impact activity** | 174 (17.2%) | 223 (27.3%) | 168 (20.9%) | 190 (19.8%) | 172 (25.7%) | 236 (16%) |
| **Little low impact activity ^a^** | 273 (27.1%) | 260 (31.8%) | 240 (29.9%) | 291 (30.4%) | 227 (33.9%) | 391 (26.6%) |
| **Moderate low impact ^a^ activity** | 317 (31.4%) | 195 (23.9%) | 241 (30%) | 267 (27.9%) | 187 (28%) | 499 (33.9%) |
| **High low impact activity ^a^** | 127 (12.6%) | 58 (7.1%) | 73 (9.1%) | 83 (8.7%) | 50 (7.5%) | 228 (15.5%) |
| **Missing low impact activity** | 118 (11.7%) | 81 (9.9%) | 82 (10.2%) | 127 (13.3%) | 33 (4.9%) | 118 (8%) |
| **No power sports** | 607 (60.2%) | 624 (76.4%) | 548 (68.2%) | 634 (66.2%) | 476 (71.2%) | 796 (54.1%) |
| **Little power sports ^b^** | 123 (12.2%) | 80 (9.8%) | 87 (10.8%) | 102 (10.7%) | 95 (14.2%) | 194 (13.2%) |
| **Moderate power sports ^b^** | 99 (9.8%) | 21 (2.6%) | 55 (6.8%) | 61 (6.4%) | 38 (5.7%) | 214 (14.5%) |
| **High power sports ^b^** | 54 (5.4%) | 2 (0.2%) | 23 (2.9%) | 23 (2.4%) | 17 (2.5%) | 138 (9.4%) |
| **Missing information** | 126 (12.5%) | 90 (11%) | 91 (11.3%) | 138 (14.4%) | 43 (6.4%) | 130 (8.8%) |
| **No morning stiffness** | 177 (17.5%) | 148 (18.1%) | 132 (16.4%) | 110 (11.5%) | 134 (20%) | 437 (29.7%) |
| **Morning stiffness <30 minutes** | 138 (13.7%) | 95 (11.6%) | 98 (12.2%) | 108 (11.3%) | 85 (12.7%) | 220 (15%) |
| **Morning stiffness 30 minutes – 1 hour** | 190 (18.8%) | 144 (17.6%) | 144 (17.9%) | 183 (19.1%) | 128 (19.1%) | 280 (19%) |
| **Morning stiffness 1-2 hours** | 154 (15.3%) | 142 (17.4%) | 133 (16.5%) | 163 (17%) | 103 (15.4%) | 171 (11.6%) |
| **Morning stiffness 2-4 hours** | 114 (11.3%) | 96 (11.8%) | 105 (13.1%) | 127 (13.3%) | 86 (12.9%) | 129 (8.8%) |
| **Morning stiffness >4 hours** | 55 (5.5%) | 50 (6.1%) | 53 (6.6%) | 69 (7.2%) | 49 (7.3%) | 69 (4.7%) |
| **Morning stiffness all day** | 55 (5.5%) | 57 (7%) | 46 (5.7%) | 65 (6.8%) | 48 (7.2%) | 59 (4%) |
| **Missing information** | 126 (12.5%) | 85 (10.4%) | 93 (11.6%) | 133 (13.9%) | 36 (5.4%) | 107 (7.3%) |
| **DAS28-esr score (SD)** | 5.1 (1.3) | 4.9 (1.4) | 5.1 (1.3) | 5.2 (1.2) | 4.8 (1.4) | 4.4 (1.4) |
| **EuroQol score (SD) [% missing]** | 52.5 (22.2) [67.8%missing] | 45.7 (21.4) [77.8% missing] | 50.7 (21.5) [74.3% missing] | 47.5 (21.1) [70.5% missing] | 47.8 (22.9) [78.2% missing] | 62.2 (21.1) [76.1%missing] |
| **HAQ score (SD) [% missing]** | 1.5 (0.7) [13%missing] | 1.7 (0.6)  [11% missing] | 1.6 (0.6)  [11.9% missing] | 1.6 (0.6)  [14.1% missing] | 1.6 (0.7)  [7.3% missing] | 1.2 (0.7) [8.6%missing] |
| **Pain level today, scale 1-10 (SD) [% missing]** | 6.2 (2.4) [12.6%missing] | 6.1 (2.5)  [9.7% missing] | 6.1 (2.4)  [10.9% missing] | 6.5 (2.2)  [13.3% missing] | 5.7 (2.6)  [5.2% missing] | 4.7 (2.8) [6.5%missing] |
| **Activity of rheumatic disease, scale 1-10 (SD) [% missing]** | 6.3 (2.4) [13.3%missing] | 6.1 (2.5)  [10.3% missing] | 6.2 (2.4)  [11.1% missing] | 6.4 (2.3)  [13.6% missing] | 5.8 (2.6)  [5.8% missing] | 4.9 (2.7) [6.9%missing] |
| **SF12 physical component score (SD) [% missing]** | 29.4 (7.9) [23.1%missing] | 28.2 (6.9) [22.3% missing] | 28.5 (7)  [22.6% missing] | 28.7 (7.1)  [24% missing] | 29.3 (8)  [17.9% missing] | 33.9 (9.7) [16.8%missing] |
| **SF12 mental component score (SD) [% missing]** | 43 (12.7) [23.1%missing] | 40.8 (12.4) [22.3% missing] | 42.4 (12.3) [22.6% missing] | 41 (11.9) [24% missing] | 43.7 (12.4) [17.9% missing] | 45.3 (12) [16.8%missing] |
| **Prednison use (%)** | 401 (39.7%) | 361 (44.2%) | 349 (43.4%) | 433 (45.2%) | 302 (45.1%) | 648 (44%) |
| **Median use (IQR) [years]** | 2.2 (0.7-6.3) | 1.4 (0.6-4.1) | 1.5 (0.6-4.3) | 1.2 (0.5-3.9) | 1.4 (0.5-4.3) | 1.1 (0.5-3.2) |
| **Methotrexate use (%)** | 599 (59.4%) | 450 (55.1%) | 471 (58.6%) | 593 (61.9%) | 386 (57.7%) | 861 (58.5%) |
| **Median use (IQR) [years]** | 4.1 (1.4-8.8) | 2.6 (1-6.5) | 2.9 (1-7.1) | 2.4 (0.8-5.6) | 3.3 (1.1-6.8) | 2.3 (0.9-4.9) |
| **Leflunomid use (%)** | 270 (26.8%) | 198 (24.2%) | 206 (25.6%) | 249 (26%) | 152 (22.7%) | 336 (22.8%) |
| **Median use (IQR) [years]** | 2.4 (0.9-5.2) | 1.4 (0.7-4.1) | 1.6 (0.7-4.3) | 1.2 (0.5-3.3) | 1.5 (0.6-3.4) | 1.3 (0.7-3.1) |
| **Sulfosalazin use (%)** | 167 (16.6%) | 123 (15.1%) | 118 (14.7%) | 149 (15.6%) | 114 (17%) | 256 (17.4%) |
| **Median use (IQR) [years]** | 3.9 (1.1-8.7) | 2.1 (0.9-6) | 2.2 (0.8-6.9) | 2.1 (0.6-5.2) | 2.5 (0.8-5.7) | 2.1 (0.7-4.3) |

ACPA: Anti-citrullinated protein antibodies; BMI: body mass index; CRP: C-reactive protein; DAS: disease activity score; DMARD: disease modifying anti-rheumatic drug, ESR: erythrocyte sedimentation rate; EuroQoL: a standardized instrument for measuring generic health status (EQ-5D), HAQ: health assessment questionnaire; IQR: interquartile range, RA: rheumatoid arthritis; SD: standard derivation, SF: Short form (health survey);

Features in red color were selected as parameters for stratified analysis.

^a^ low: <30 min daily walking / cycling, Moderate: 30-60 min daily walking / cycling, high: ≥60 min daily walking / cycling

^b^ low : <60 min power sports per week, Moderate: 1-2 h power sports per week, high:  ≥2 h power sports per week
