# Supplementary material for: Patient groups in Rheumatoid arthritis identified by deep learning respond differently to biologic or targeted synthetic DMARDs
Source: PLoS Comput Biol. 2023 Jun 2;19(6):e1011073. doi: 10.1371/journal.pcbi.1011073 (PMC10266686; doi:10.1371/journal.pcbi.1011073)
Supplement: S6 Table — (DOC) [file pcbi.1011073.s018.doc]

**S6 Table.** Clusters of patients with a rather low RA disease burden and with a tendency towards seropositivity and a higher proportion of women

|  | **Cluster**  **(n=1481)** | **Cluster**  **(n=1142)** | **Cluster**  **(n=688)** | **Cluster**  **(n=1009)** | **Cluster**  **(n=1145)** |
| --- | --- | --- | --- | --- | --- |
| **Mean age (SD) [years]** | 52 (13.9) | 53.7 (13.6) | 52.1 (14.2) | 54.8 (14.2) | 53.7 (14) |
| **Women (%)** | 977 (66%) | 750 (65.7%) | 688 (100%) | 1009 (100%) | 1145 (99.9%) |
| **Men (%)** | 504 (34%) | 392 (34.3%) | (0%) | (0%) | (0%) |
| **Median RA duration (IQR) [% missing]** | 4.2 (2-9) [1.6%missing] | 4.6 (2.3-10.3) [1.6% missing] | 4.9 (2.4-10.2) [2.2%missing] | 6.6 (2.7-15.3) [2.1% missing] | 4.4 (2.1-8.6) [2.4%missing] |
| **Rheumatoid factor negative (%)** | 512 (34.6%) | 0% | (0%) | (0%) | (0%) |
| **Rheumatoid factor positive (%)** | 917 (61.9%) | 1084 (94.9%) | 653 (94.9%) | 950 (94.2%) | 1085 (94.7%) |
| **Missing information** | 52 (3.5%) | 58 (5.1%) | 35 (5.1%) | 59 (5.9%) | 61 (5.3%) |
| **ACPA negative** | 466 (31.5%) | 173 (15.2%) | 106 (15.4%) | 132 (13.1%) | 178 (15.5%) |
| **ACPA positive** | 568 (38.4%) | 679 (59.5%) | 406 (59%) | 621 (61.6%) | 711 (62%) |
| **Missing information** | 447 (30.2%) | 290 (25.4%) | 176 (25.6%) | 256 (25.4%) | 257 (22.4%) |
| **No family history of rheumatic diseases** | 594 (40.1%) | 525 (46%) | 295 (42.9%) | 431 (42.7%) | 600 (52.4%) |
| **Family history of rheumatic diseases** | 349 (23.6%) | 246 (21.5%) | 165 (24%) | 267 (26.5%) | 197 (17.2%) |
| **Missing information** | 538 (36.3%) | 371 (32.5%) | 228 (33.1%) | 311 (30.8%) | 349 (30.5%) |
| **Non-smoker** | 246 (16.6%) | 188 (16.5%) | 144 (20.9%) | 253 (25.1%) | 260 (22.7%) |
| **Current smoker** | 357 (24.1%) | 250 (21.9%) | 119 (17.3%) | 187 (18.5%) | 224 (19.6%) |
| **Mean no. of years smoking (SD)** | 24.8 (11.7) | 26.8 (11.1) | 26.6 (11) | 28.8 (11.5) | 26.9 (10.2) |
| **≤1 package per day** | 193 (13%) | 153 (13.4%) | 63 (9.2%) | 101 (10%) | 123 (10.7%) |
| **>1 package per day** | 28 (1.9%) | 136 (11.9%) | 5 (0.7%) | 9 (0.9%) | 16 (1.4%) |
| **Former smoker** | 140 (9.5%) | 14 (1.2%) | 62 (9%) | 126 (12.5%) | 156 (13.6%) |
| **Missing smoking** | 738 (49.8%) | 551 (48.3%) | 363 (52.8%) | 443 (43.9%) | 506 (44.2%) |
| **Mean BMI (SD) [% missing]** | 24.8 (4.5) [7.5%missing] | 25.3 (4.8) [6.5% missing] | 24.5 (4.8) [6.4% missing] | 23.9 (4.4) [5.7% missing] | 25.5 (5.2) [7.9% missing] |
| **No low impact activity** | 183 (12.4%) | 51 (4.5%) | 40 (5.8%) | 56 (5.6%) | 60 (5.2%) |
| **Little low impact activity ^a^** | 363 (24.5%) | 244 (21.4%) | 155 (22.5%) | 230 (22.8%) | 248 (21.6%) |
| **Moderate low impact ^a^ activity** | 477 (32.2%) | 435 (38.1%) | 263 (38.2%) | 425 (42.1%) | 454 (39.6%) |
| **High low impact activity ^a^** | 297 (20.1%) | 279 (24.4%) | 153 (22.2%) | 239 (23.7%) | 249 (21.7%) |
| **Missing low impact activity** | 161 (10.9%) | 133 (11.7%) | 77 (11.2%) | 59 (5.9%) | 135 (11.8%) |
| **No power sports** | 627 (42.3%) | 361 (31.6%) | 75 (10.9%) | 359 (35.6%) | 429 (37.4%) |
| **Little power sports ^b^** | 200 (13.5%) | 182 (15.9%) | 236 (34.3%) | 170 (16.9%) | 156 (13.6%) |
| **Moderate power sports ^b^** | 287 (19.4%) | 276 (24.2%) | 108 (15.7%) | 260 (25.8%) | 266 (23.2%) |
| **High power sports ^b^** | 196 (13.2%) | 189 (16.6%) | 168 (24.4%) | 162 (16.1%) | 159 (13.9%) |
| **Missing information** | 171 (11.6%) | 134 (11.7%) | 101 (14.7%) | 58 (5.8%) | 136 (11.9%) |
| **No morning stiffness** | 519 (35%) | 422 (37%) | 303 (44%) | 477 (47.3%) | 426 (37.2%) |
| **Morning stiffness <30 minutes** | 235 (15.9%) | 203 (17.8%) | 124 (18%) | 189 (18.7%) | 189 (16.5%) |
| **Morning stiffness 30 minutes – 1 hour** | 238 (16.1%) | 198 (17.3%) | 100 (14.5%) | 154 (15.3%) | 195 (17%) |
| **Morning stiffness 1-2 hours** | 136 (9.2%) | 93 (8.1%) | 46 (6.7%) | 59 (5.9%) | 99 (8.6%) |
| **Morning stiffness 2-4 hours** | 90 (6.1%) | 57 (5%) | 22 (3.2%) | 33 (3.3%) | 58 (5.1%) |
| **Morning stiffness >4 hours** | 44 (3%) | 27 (2.4%) | 11 (1.6%) | 18 (1.8%) | 25 (2.2%) |
| **Morning stiffness all day** | 60 (4.1%) | 17 (1.5%) | 11 (1.6%) | 13 (1.3%) | 16 (1.4%) |
| **Missing information** | 159 (10.7%) | 125 (11%) | 71 (10.3%) | 66 (6.5%) | 138 (12%) |
| **DAS28-esr score (SD)** | 3.9 (1.3) | 4.2 (1.4) | 3.7 (1.1) | 3.7 (1.1) | 4.2 (1.3) |
| **EuroQol score (SD) [% missing]** | 67.4 (17.9) [67.9%missing] | 70.4 (15.2) [63.7% missing] | 71.3 (16) [65% missing] | 72.9 (13.5) [58.6% missing] | 68.3 (17.7)  [56.2% missing] |
| **HAQ score (SD) [% missing]** | 0.8 (0.6) [11.3%missing] | 0.7 (0.5)  [11.6% missing] | 0.7 (0.5)  [11.5% missing] | 0.7 (0.5)  [8.2% missing] | 0.8 (0.6)  [12.1% missing] |
| **Pain level today, scale 1-10 (SD) [% missing]** | 3.9 (2.6) [10%missing | 3.7 (2.5)  [10.6% missing] | 3.2 (2.3)  [10% missing] | 3.1 (2.2)  [6.9% missing] | 3.8 (2.6)  [11.6% missing] |
| **Activity of rheumatic disease, scale 1-10 (SD) [% missing]** | 4.2 (2.6) [10.4%missing] | 4.0 (2.5)  [11.2% missing] | 3.7 (2.3)  [11.2% missing] | 3.6 (2.2)  [7.3% missing] | 4.2 (2.5)  [12.2% missing] |
| **SF12 physical component score (SD) [% missing]** | 37.6 (9.7) [18.4%missing] | 38.7 (9.5)  [17.1% missing] | 40.8 (9)  [18.9% missing] | 40.4 (9.1)  [17.6% missing] | 38.5 (9.7)  [20.4% missing] |
| **SF12 mental component score (SD) [% missing]** | 46.4 (11.7) [18.4%missing] | 48.6 (10.7) [17.1% missing] | 48.1 (11.2)  [18.9% missing] | 49.7 (10.3) [17.6% missing] | 46.9 (11.4)  [20.4% missing] |
| **Prednison use (%)** | 570 (38.5%) | 458 (40.1%) | 284 (41.3%) | 476 (47.2%) | 524 (45.7%) |
| **Median use (IQR) [years]** | 1 (0.5-2.1) | 1 (0.5-2.5) | 1.1 (0.5-2.7) | 1.2 (0.5-3.4) | 1 (0.5-2.5) |
| **Methotrexate use (%)** | 907 (61.2%) | 747 (65.4%) | 460 (66.9%) | 736 (72.9%) | 815 (71.1%) |
| **Median use (IQR) [years]** | 1.1 (0.6-2.7) | 1.6 (0.7-3.7) | 1.4 (0.7-3.4) | 2.1 (0.8-5) | 1.6 (0.7-4) |
| **Leflunomid use (%)** | 314 (21.2%) | 271 (23.7%) | 164 (23.8%) | 284 (28.2%) | 308 (26.9%) |
| **Median use (IQR) [years]** | 1 (0.5-2.1) | 1.2 (0.6-2.8) | 1.3 (0.7-3.3) | 1.7 (0.8-3.8) | 1.1 (0.6-2.7) |
| **Sulfosalazin use (%)** | 235 (15.9%) | 183 (16%) | 136 (19.8%) | 229 (22.7%) | 225 (19.6%) |
| **Median use (IQR) [years]** | 1.1 (0.6-2.6) | 1.6 (0.7-3.6) | 1.4 (0.8-3.4) | 2 (0.8-5.1) | 1.6 (0.7-3.4) |

ACPA: Anti-citrullinated protein antibodies; BMI: body mass index; CRP: C-reactive protein; DAS: disease activity score; DMARD: disease modifying anti-rheumatic drug, ESR: erythrocyte sedimentation rate; EuroQoL: a standardized instrument for measuring generic health status (EQ-5D), HAQ: health assessment questionnaire; IQR: interquartile range, RA: rheumatoid arthritis; SD: standard derivation, SF: Short form (health survey);

Features in red color were selected as parameters for stratified analysis.

^a^ low: <30 min daily walking / cycling, Moderate: 30-60 min daily walking / cycling, high: ≥60 min daily walking / cycling

^b^ low : <60 min power sports per week, Moderate: 1-2 h power sports per week, high:  ≥2 h power sports per week
