# Supplementary material for: Patient groups in Rheumatoid arthritis identified by deep learning respond differently to biologic or targeted synthetic DMARDs
Source: PLoS Comput Biol. 2023 Jun 2;19(6):e1011073. doi: 10.1371/journal.pcbi.1011073 (PMC10266686; doi:10.1371/journal.pcbi.1011073)
Supplement: Attachment — Submitted filename: Response_Letter_Burden_PCOMPBIOL-D-21-01954.R1.docx [file pcbi.1011073.s020.docx]

Departement Chemie und Angewandte Biowissenschaften
Institut für Pharmazeutische Wissenschaften

ETH Zürich

Dr. Theresa Burkard

Vladimir-Prelog-Weg 1-5/10

CH 8093 Zürich

theresa.burkard@pharma.ethz.ch

Prof. Feilim Mac Gabhann

Prof. Jason Papin

Editors-in-chief

PLOS Computational Biology

September 12, 2022

**Revision letter concerning manuscript PCOMPBIOL-D-21-01954.R1**

**"Patient Groups in Rheumatoid Arthritis Identified by Deep Learning Respond Differently to Biologic or Targeted Synthetic DMARDs"**

Dear Prof. Mac Gabhann,

Dear Prof. Papin,

We thank you and the reviewers for the positive feedback and comments.

Below we provide point-by-point responses to the individual comments. Additionally, all changes to the text have been marked in the accompanying manuscript using track change.

We hope that we were able to address all comments to your satisfaction and we would be pleased to see our manuscript published in the Journal “PLOS Computational Biology”.

Sincerely,

Theresa Burkard

**REVIEWER COMMENTS:**

**Reviewer #1:**

In this work, the authors report on a study that combines machine learning with comparative effectiveness analysis aimed at identifying which b/tsDMARDs would work “better” or “less” for patients subgroups. Two separate outcomes were used (1) non-response and (2) treatment response within a window of 15 months. For (1) it is a binary outcome defined as discontinuation of b/tsDMARD after physician’s recording of non-response. For (2) it is also a binary outcome defined as reduction in DAS28-esr by >=20%.  Additionally, the dataset included 3 classes of treatments (1) TNF inhibitor, (2) non-TNF inhibitor and (3) tsDMARD where each of the classes had a set of drugs associated with them. The reference group for the class effect analysis was the TNF inhibitor.
 
The authors used AnyNets (neural network architecture  <http://ceur-ws.org/Vol-2926/paper2.pdf>), specifically the autoencoder version to learn a latent representation from the encodings of the “present” features. These latent representations are used in a deep embedded clustering routine that involves a composite loss function. Clustering was done for 3, 4 and 5 predefined clusters. Additionally, they added “the time since measurement” to the features and repeated the 3, 4, 5 clustering to obtain a total of 24 clusters. Given that clusters were having similar features/characteristics, the authors regrouped them into 5 patient groups (based on the authors’ heuristics) and then extracted the most important features defining the groups for conducting a stratified analysis.
 
The first analysis used the 24 clusters to compare effectiveness of b/tsDMARDs using Cox proportional hazard regression to estimate hazard ratios of treatment no-response and treatment response separately. The results were compared among clusters belonging to same group (i.e. the groups they established above). Significant results present in the maximum number of clusters of the same group were reported. The same analysis was further repeated but this time using stratified groups based on the important features they determined earlier.
 
Overall, the study is interesting and informative and reports on “ad-hoc” approach that combines ML with regression analysis to establish treatment effect estimation.

The authors should address the following comments:
 
1- In many places in the writing, the authors would refer to “regrouping” or “selecting most distinctive features” etc. without providing details on the heuristics for selection. For example, what was the bases for regrouping in 5 groups, and how were the “few important” features selected for the validation analysis? It is worth describing the workflow; did the authors use comparisons/statistical tests for these decisions.

Response:

Our approach to group the clusters was indeed heuristic, it was not perfect but pragmatic. We visually inspected the 24 clusters and compared them (column by column) by marking their most extreme values. The use of ANOVA or similar testing would not be expedient in this situation because a significant result would tell us that at least two values are different across the 24 different clusters which would need further ad hoc testing to answer which values were considered different, but this would have implied a lot of significance testing, given 24 clusters and 43 or 52 variables (in run #1 or run #2, respectively).

However, to be clearer about our approach, we have now added a description of the workflow in the manuscript as well as the reason why we did not perform significance testing.

Line 185 ff.

“Because findings from individual clusters may be chance findings and each cluster was similar in patient characteristics to at least one other cluster, we grouped clusters manually according to most distinct patient characteristics. The grouping was based upon visual comparison of patient characteristics. Most extreme characteristics (i.e. highest and lowest frequencies/values) were marked, and by „putting these together“, groups were formed. An example with few clusters and patient characteristics is given in Supplementary File S5. Significance testing of patient characteristics using ANOVA (Analysis of Variance) and subsequent Least Significance Difference tests was not feasible given the number of clusters and variables.”

We further added an example to the Supplementary Files (Supplementary File S5).

**Supplementary File S5: Example of approach how cluster groups were formed.**

All columns were put side by side (in this example only a fraction of columns and pre-selected characteristics are seen). Most extreme values were marked. In a further step, columns were re-shuffled to put similar columns next to each other. Groups were formed.

**Supplementary Figure S8.** Selected patient characteristics of selected clusters with extreme values marked in red

Men

Seronegative patients with lowest use of prednisone and a tendency towards a higher proportion of women

Seronpositive women with a rather high disease burden and long disease duration

Patients with a rather low disease burden and a tendency towards seropositivy and a higher proportion of women

Patients with highest use of csDMARDs and prednisone, a tendency towards no family history of RD and a higher proportion of women

**Supplementary Figure S9.** Selected patient characteristics of selected clusters with extreme values marked (in red) and columns re-shuffled to put similar clusters next to each other.

2- The use of AnyNet to cluster patients is an interesting choice. The authors should report on a baseline vanilla “k-means” or “hierarchical clustering” and compare the obtained groups. How many of the generated groups and subsequent analysis (of significant effects) still hold?

Response:

We thank the reviewer for this suggestion. While it is indeed desirable to have several clustering techniques yielding similar results, we did not obtain separated clusters when employing k-means clustering. Since we were not able to form groups from these clusters, we refrained from further comparative effectiveness analyses. Patient data unfortunately has missing data and the algorithm we used (AnyNets) was designed to handle missing data. It outperformed other algorithms in this situation which we had already stated as such in the Methods section (lines 147 ff).

We added this post-hoc analysis of k-means clustering to the methods section and discussion section as follows.

Line 162 ff.

“In post-hoc analyses to compare obtained clusters, k-means clustering without deep learning was performed. We did not obtain separated clusters (Supplementary File S3).”

Line 367 ff.

“Our post-hoc approach using k-means clustering did not yield separate clusters which suggests that deep learned clusters may be superior to k-means clusters when working with missing data which is usually the case in health care data.”

**Supplementary File S3: Post-hoc analyses using k-means clustering**

**Supplementary Figure S2.** Results of K-means clustering using specification #1 requesting 3 clusters.

**Supplementary Figure S3.** Results of K-means clustering using specification #1 requesting 4 clusters.

**Supplementary Figure S4.** Results of K-means clustering using specification #1 requesting 5 clusters.

**Supplementary Figure S5.** Results of K-means clustering using specification #2 requesting 3 clusters.

**Supplementary Figure S6.** Results of K-means clustering using specification #1 requesting 4 clusters.

**Supplementary Figure S7.** Results of K-means clustering using specification #1 requesting 5 clusters.

3- Did the authors consider the use and fitting of an “exposure model” that is useful for propensity score matching. In others words, the stratification would be based on the propensity scores generated from the exposure model.

Response:

To form patient groups according to strata obtained through estimating propensity score values of receiving b/tsDMARD treatment may be a valuable approach. This approach was indeed discussed, however, since appropriate machine learning tools were shown to be superior in finding patterns in data (i.e. distinct patient groups) when compared to traditional statistical methods, we chose the approach to employ deep learned patient clusteres. (Bzdok, D., Altman, N. & Krzywinski, M. Statistics versus machine learning. *Nat Methods* **15**, 233–234 (2018). https://doi.org/10.1038/nmeth.4642)

4- The study does not deal with the dynamic (i.e. temporal) aspect of patients profiles during the 15-month window. Did the authors plan to incorporate dynamic information? This is something to discuss even if it is not planned for this study.

Response:

To include temporal information may be important if measured confounding variables are fast changing over time. However, there are no confounding variable in this study question that fall in this category. While markers of pain, disability and disease activity are fast changing and may be considered by some to be confounding variables, they really lie on the causal pathway and therefore are mediators between exposure and outcome. Including them in the regression model will cause a bias towards the null (i.e. adjusting for the outcome).

To include this in the discussion of the manuscript, we now touch upon the use of confounders in the limitation section as follows:

Line 351 ff.

“Finally, we were not able to adjust our analyses for confounding variables like obesity because of the small sample size. However, crude and age/sex adjusted HR were identical for most of our results. Thus, we do not expect an influence on our results from other measured or non-measured confounding. While time-varying confounder adjustment may be desirable in comparative effectiveness analyses in rheumatologic conditions, markers of pain, disability, and disease activity often lie on the causal pathway between exposure and outcome and should therefore not be included in the model.”

5- There are multiple studies that use ML for treatment effect estimation (i.e. individualised treatment effects). A pointer to the literature that is heavily researched in the last couple of years would further contextualise this work.

Response:

We agree with the reviewer that there is a strong dynamic in the literature about machine learning and treatment effect estimation.

While there may be more literature in other diseases, or in the use of non-deep learning machine learning methods, to include this type of literature in our discussion would go beyond the scope of our manuscript which focuses on rheumatic diseases and data driven clusters. To our knowledge this is the first clustering model that is being evaluated by strong epidemiologic standards, thus, no literature exists with which we could perform a 1:1 comparison. However, when we re-assessed our literature research, we found two recently performed cluster analyses among rheumatoid arthritis patients which we have now added.

Please find the amended introduction and discussion as follows:

Line 358 ff.

“Obtained clusters in three recent studies using k-means clustering to identify RA phenotypes were mainly driven by disease activity and comorbidities, sex and comorbidities, and disease activity and health care costs, respectively.(11–13) In our study, we did not assess comorbidities or health care costs, but we can confirm the importance of disease activity and sex, and we additionally identified seropositivity, disease duration, and other RA medication use at b/tsDMARD start as important variables for differentiating between clusters. It seems that the RA population in the cited studies were rather diverse and not linked to RA treatment, whereas we clustered our patients at b/tsDMARD start. However, despite using different clustering techniques and patient populations, the overlap between clusters is promising.”

New references:

12.	Jung S, Park K s, Kim K j, Min Jung S, Park KS, Kim KJ. Clinical phenotype with high risk for initiation of biologic therapy in rheumatoid arthritis: a data-driven cluster analysis Unsupervised clustering of RA / S.M. Jung et al [Internet]. Vol. 39, Clinical and Experimental Rheumatology. 2022. Available from: www.r-project.org

13.	Mars N, Kerola AM, Kauppi MJ, Pirinen M, Elonheimo O, Sokka-Isler T. Cluster analysis identifies unmet healthcare needs among patients with rheumatoid arthritis. Scand J Rheumatol. 2022;51(5):355–62.

6- Minor comments : consider adding a visual/plot on how many times the reported variables were significant across all clusters (within groups)

Response:

We thank the reviewer for this comment. We are sorry that we cannot accommodate this suggestion because we did not perform statistical testing. We kindly refer the reviewer to the response of reviewer comments No. 1) where we state the reasons why statistical testing was not expedient in our study when we identified the five groups among the 24 clusters.

**Reviewer #2:**

I read with interest this paper on the identification of patient groups in rheumatoid arthritis by deep learning. This paper is a follow-up to a previous paper published in PLOS One in 2021 and referenced as reference 7 in this paper. The results only are of interest for 2 specific patient subgroups and rest on quite the usual criteria for response to treatment; however the approach warrants publication. Here are some comments to improve the paper.

1. Abstract results. The formulation of the observations at the end of the sentences makes it a little difficult to understand the results. Is it possible to reformulate?

Response:

Given the restraint of number of words in the abstract (a maximum of 300 words) while complying with the guidance of STROBE (**STrengthening the Reporting of OBservational studies in Epidemiology)** guidance to give a balanced summary of results and to give those in numbers in the abstract, we are afraid it is not possible to re-formulate the results, unless the reviewer has a suggestion on how to implement this.

2. Author summary page 3. I think it would be more useful to describe the five are a patient groups or at least to cite the elements defining these patient groups which are gender previous treatment and disease burden.

Response:

We expanded the author summary and included a brief description of the patient groups and their treatment responses.

Please find the amended Author Summary as follows.

“Rheumatoid arthritis (RA) is an auto-immune disease affecting the joints of the body. RA is subject to poor treatment response to advanced antirheumatic therapy. While previous studies have used machine learning techniques to identify different RA patient populations, no study has used these populations to evaluate potentially different treatment response of advanced RA treatments such as tumor necrosis factor inhibitors. Using machine learning, we identified five distinct RA patient groups which mainly differed by sex, disease burden/duration, and concomitant traditional RA treatment use (i.e. prednisone, methotrexate). Patients with high frequency of use of traditional RA treatment use at advanced RA treatment initiation, male patients, as well as patients with a lower disease burden responded better to tocilizumab than to adalimumab. Furthermore, seronegative women without use of prednisone at advanced RA treatment initiation as well as seropositive women with a higher disease burden and longer disease duration had a higher risk of non‑response with golimumab than with adalimumab. The results are a step towards personalizing treatment and shall encourage other researchers to embrace machine learning techniques to improve treatment response in RA and other disease areas.”

3. Methods, data source p5. The fact that the data refers to a very long period, 1999 to 2018, may have influenced the results since patient and physician expectations as well as the treatment armamentarium has changed a lot over this period. Could the authors please comment?

Response:

We agree with the reviewer that the availability of b/tsDMARDs has changed over the years. This is of particular importance when performing traditional epidemiologic research. However, our basis for comparative effectiveness analyses were clusters found by a machine learning approach given patient characteristics at (any) b/tsDMARD initiation. Thus, which b/tsDMARD was initiated does not matter in our approach, especially because the particular b/tsDMARD agent was not a feature for the clustering. Moreover, a study performed in SCQM data in 2013 suggested that patient characteristics at bDMARD initiation has not changed over time (Zufferey et al. Joint Bone Spine 2013). Hence, we expect no bias resulting from the differing availability of b/tsDMARDs over time.

Finally, in order for patient and physician expectation to bias our results, they would need to be differentially distributed between treatment groups. There is no reason for a difference in patient or physician expectation between those receiving TNF inhibitors (e.g. adalimumab) or other b/tsDMARDs as first therapy choice when escalating to a b/tsDMARD since EULAR guidance does not commend one over another.

We summarised this as a strength in the discussion as follows:

Line 326 ff.

“Finally, since the particular b/tsDMARD agent was not a feature in the clustering and a study in SCQM suggested no changes in patient characteristics at bDMARD initiation over time,(22) our cluster results have not been biased from time trends. Furthermore, we can also likely rule out bias from patient and physician expectations because there is no reason for a differentially distributed expectation between individual b/tsDMARD treatments since EULAR RA treatment guidance does not commend one over another.(2)”

New reference:

22.	Zufferey P, Dudler J, Scherer A, Finckh A. Disease activity in rheumatoid arthritis patients at initiation of biologic agents and 1 year of treatment: Results from the Swiss SCQM registry. Joint Bone Spine [Internet]. 2013;80(2):160–4. Available from: http://dx.doi.org/10.1016/j.jbspin.2012.05.003

4. Exposures p6. It is surprising to me that the non TNFis are grouped in the methods, whereas they are reported separately in the results where the main results actually concern only Toci and Goli, representing only a small portion of the patients. Please comment.

Response:

We are not quite sure we understand this comment, but we slightly rephrased a paragraph in the methods section from which the confusion may have arisen.

Please find the amended Exposures paragraph as follows:

Line 120 ff.

“The exposure was defined as the first bDMARD (TNF inhibitors: adalimumab, certolizumab pegol, etanercept, infliximab, golimumab; non-TNF inhibitors: abatacept, rituximab, tocilizumab), or tsDMARD (baricitinib, tofacitinib). Comparative effectiveness analyses were performed on a class effect basis and on an individual b/tsDMARD basis. The reference group in the class effect analyses was TNF inhibitors, and the reference group in the individual b/tsDMARD analyses was adalimumab.”

5. Methods page 8 statistics. When describing the patient demographics and lifestyle factors on the top of page 8, please give the detail of which lifestyle factors were explored here.

Response:

Supplementary File S4 state in detail all features. However, we now added in brackets to this paragraph which lifestyle factors were available, i.e. physical activity, body mass index, and smoking, as follows:

Line 165 ff.

“Features for patient clustering included patient demographics and life-style factors (e.g., physical activity, body mass index, smoking) that were considered time-invariant and thus captured using an ever before lookback window.”

6. Data analysis p9. I personally found the second paragraph on page 9 starting with stratification very complex to understand. Is it possible to simplify it?

Response:

We rephrased this paragraph and thereby tried to simplify it as follows.

Line 195 ff.

“When clustering patients, we use the strength of machine learning techniques and include many features (i.e. patient characteristics). Using these results in clinical decision-making implies that all characteristics would need to be assessed at the moment of decision-making which is not feasible. Therefore, we further employ stratified analyses which make results applicable to clinical practice. Stratification means that patients are categorized according to few (most important) characteristics only. and is the preferred method to validate results obtained through clustering. In this study, we used the strength of clustering to set the basis to define the most distinct patient characteristics that set the different groups apart, which would not have been possible otherwise. Categories of these most distinct features were then derived as parameters for the stratified (validation) analyses (Figure 2).”

7. Statistics page 10 first paragraph. It seems there was no external validation patient group. Is this correct? My understanding of artificial intelligence is that it is of key importance to validate the results on an external data set. Please consider this.

Response:

It is correct that there was no external validation of our results. We mention in the conclusion that validation of our results in other cohorts is needed in the future to externally validate our results, but we now added a sentence to the limitation section stating this shortcoming as follows.

Line 346 ff.

“A further limitation of our study is that we did not have an external cohort at hand to further validate our results externally. Thus, caution is warranted when using our findings in clinical practice.”

8. Page 10 results. The authors obtained 24 clusters. I believe there must have been a lot of overlap between the clusters. Is this the case? Please comment.

Response:

It is correct, that there was a lot of overlap between clusters. This overlap was indeed desired and needed to obtain robust results in our study. We wanted similar, but not the same clusters. Thus, if these similar clusters yield the same results in a comparative effectiveness analyses, chance findings through overfitting are prevented. Thus, we can consider our results robust, i.e. findings insensitive to minor variation. We think of our similar but not identical clusters variation like a type of re-sampling.

Therefore, the Features section (lines 180 ff.) states that the plethora of cluster runs increased robustness of our findings. And the Data analyses section (lines 188 ff.) state that chance findings were prevented by assessing similar clusters.

Finally, the strength statement about this was already quite extensive and therefore we only slightly amended this part as follows:

Line 313 ff.

“Further strength include the use of several clustering runs that obtained 2 dozen different clusters which we grouped according to similar patient characteristics. We admit that this process made the study more complex and potentially less reproducible because the visual inspection of clusters to form distinct groups may lead to different grouping depending on who is inspecting the clusters. A continuation of this work could include the automation of grouping of clusters through learning of a meta-classifier for example. However, such a program does not exist yet. While grouping of clusters may take away some information on clusters that machine learning techniques provided, results also need to be robust. The comparison of comparative effectiveness results among groups of similar clusters made our results less prone to chance findings. Having assessed similar but not identical clusters can be considered like a type of re-sampling. The overlap between clusters was indeed desired and needed to obtain robust results insensitive to minor variation. This was important because sample size of individual clusters was small.”

9. Five patient groups described page 10. It seems that the patient groups are defined mainly on gender and previous drug use. This is not very original. It is unfortunate that not more data were entered into the clusters. Could the authors please comment?

Response:

Supplementary File S4 lists in detail all features used to obtain the clusters, which were plentiful (43 or 52 variables, in run #1 or run #2, respectively). We included all features that we deemed relevant and that we know were reliably captured. Thus, the answer to the question, would more variables change our clusters, is “it may depend on the variables”. While very advanced clustering technique through deep learning yielded strong and well separated clusters in our study unlikely to be changed by different feature inputs because they further yielded similar clusters in two different clustering runs, three other studies have found that comorbidities were of importance. We did not think that the self-reported comorbidity information in SCQM was reliable enough due to under-reporting. Thus, adding comorbidity information may have actually corrupted our deep learning models.

To comment on the groups being solely separated by sex and concomitant drug use. Our work confirms previous findings, and additionally suggested three other groups detected through deep learned clusters. Moreover, the addition of our work is that we were able to suggest strata including more than one characteristic. To try and combine all variables into packages of 2 characteristics yields 2^n^ possibilities (if there are n variables), if wanting to make combinations of 3, 3^n^, and so on. This type of research is first, not feasible and second, subject to multiple testing.

10. Drug use top of page 11. Could the authors comment if the use of the two conventional drugs was concomitant to the biologic, or was previous use. This is unclear from the text.

Response:

We apologize that this information was not 100% clear. The Methods section states that “Additional features such as other medication use (e.g., conventional synthetic [cs] DMARDs) were assessed at cohort entry” (line 169 ff.). This information is repeated in Supplementary File S4 which states in detail all used features. Thus, the drug use was concomitant at the time point of starting the b/tsDMARD whose initiation led to the inclusion of the patient into the study.

We slightly amended the result section to clarify this part as follows.

Line 233 ff.

“The first group comprised clusters with highest frequencies of individual csDMARD and prednisone use (≥75%, ≥31%, ≥19%, and ≥47% of patients used methotrexate, leflunomide, and sulfasalazine, and prednisone, respectively, at b/tsDMARD initiation), and with a tendency towards seropositivity and no family history of rheumatic diseases.”

11. Smoking page 11. When was the smoking data collected ? Please clarify.

Response:

This information can be found in Supplementary File S4 which states in detail all used features. This file states that the information was obtained from an ever before lookback window. This information was now added to the result section as follows.

Line 239 ff.

“The second group comprised clusters of a majority of men (>99%) which further displayed the highest frequency of smoking and mean body mass index (both assessed from an ever before lookback window).”

12. Response criteria. Using a change of DAS is not stringent. Why was this outcome chosen?

Response:

We agree that a change in DAS is not a stringent outcome, but only an approximation of treatment response. We have chosen this approximation for treatment response because it was the only way to assess treatment response at all (with a sufficient sample size at times but not always). We have added this limitation to the Discussion section as follows.

Line 348 ff.

“Furthermore, to assess a change in DAS is not a stringent outcome and can only be considered an approximation of treatment response. However, it allowed us to increase sample size without which an investigation in this direction would not have been possible.”

13. Disease activity and disease burden defined bottom of page 11 as predicted as outcomes. There is some circularity here since of course patients who do not do well will tend to not do well. Please comment.

Response:

In this part of the manuscript, we define the parameter for the validation process. One group consisted indeed of patients with high disease burden/duration, seropositivity, as found by the deep learned clustering. The hope would have been that the analysis would have revealed a b/tsDMARD that was superior in the comparative effectiveness analyses in these patients. Unfortunately, we only observed a significant non-response result. However, we are of the opinion that this may have been due to the small number of patients not allowing us to assess a good treatment response in these patients.

14. Results page 12 on the 2 drugs of interest, Toci and Goli. How many patients were in fact in these drug clusters, I found this information in the table but please add it to the text.

Response:

The reviewer has found the number of outcomes in the tables, not the number of patients which is usually not shown in hazard regression modelling, because the denominator is person-time, not number of persons. However, we have added the number of outcomes to the result section as follows.

Line 264 ff.

“Clusters in the first group (i.e., high frequency of use of csDMARDs and prednisone) responded well to tocilizumab (significant HRs of 2.55-2.67, count of outcomes: 6-11, depending on the cluster). Clusters in the second group (i.e., men) also responded well to tocilizumab (significant HRs of 6.78-8.54, count of outcomes: 5-7, depending on the cluster). Clusters in the third group (i.e., seronegative patients with a tendency towards low use of prednisone and a higher proportion of women) as well as clusters in the fourth group (i.e., mainly seropositive women with high disease burden and long duration) had a high risk of non‑response with golimumab (significant HRs of 2.10-2.53 and of 2.45-4.54, count of outcomes: 19-24 and 8-12, respectively, depending on the clusters).  Clusters in the fifth group (i.e., patients with low disease burden and with a tendency towards seropositivity and a higher proportion of women) responded well to golimumab (significant HRs of 2.56-3.15, count of outcomes: 5-9, depending on the cluster) and tocilizumab (significant HRs of 3.39-6.43, depending on the cluster).”

15. Discussion page 15. The authors briefly commented on the inclusion of comorbidities – what woul that change?

Response:

While comorbidities are capture in SCQM, they are self-reported by the patient. Thus, we did not consider these variables reliable (most comorbidities have an unnaturally low prevalence). On the question of “change”, we kindly refer the reviewer to the answer of reviewer’s comment No. 9.

16. Tables and figures. I suggest to add a figure with the percentage of response to adalimumab versus competitors for the main comparisons since currently it is very difficult to get an absolute you a response rather than a relative view of response.

Response:

Since we estimated hazard ratios which depend on the follow-up time, it is right to add percentages (as it may be done when estimating odds ratios). We are sorry that we cannot comply with the reviewer´s suggestion.

17. Tables are very busy and difficult to read is there a way to simplify them? Perhaps be regrouping main significant results?

Response:

We thank the reviewer for this suggestion but would like to keep our style of tables which include the number of outcomes, as well as crude and adjusted hazard ratios as requested by STROBE guidance.

18. Minor comment. The word leflunomide takes an E and must be corrected throughout the paper.

Response:

We amended this typo throughout the manuscript.
